# Supplementary material for: THBS1-producing tumor-infiltrating monocyte-like cells contribute to immunosuppression and metastasis in colorectal cancer
Source: Nat Commun. 2023 Sep 25;14:5534. doi: 10.1038/s41467-023-41095-y (PMC10520015; doi:10.1038/s41467-023-41095-y)
Supplement: Supplementary file 1 — Supplementary Information [file 41467_2023_41095_MOESM1_ESM.pdf]

Supplementary Information

THBS1-producing tumor-infiltrating monocyte-like cells contribute to immunosuppression and metastasis in colorectal cancer

Mayuki Omatsu, Yuki Nakanishi, Kosuke Iwane, Naoki Aoyama, Angeles Duran, Yu Muta, Anxo Martinez-Ordoñez, Qixiu Han, Nobukazu Agatsuma, Kenta Mizukoshi, Munenori Kawai, Go Yamakawa, Mio Namikawa, Kensuke Hamada, Yuichi Fukunaga, Takahiro Utsumi, Makoto Sono, Tomonori Masuda, Akitaka Hata, Osamu Araki, Munemasa Nagao, Takaaki Yoshikawa, Satoshi Ogawa, Yukiko Hiramatsu, Motoyuki Tsuda, Takahisa Maruno, Toshiaki Kogame, Hiroaki Kasashima, Nobuyuki Kakiuchi, Masahiro M. Nakagawa, Kenji Kawada, Masakazu Yashiro, Kiyoshi Maeda, Yasuyuki Saito, Takashi Matozaki, Akihisa Fukuda, Kenji Kabashima, Kazutaka Obama, Seishi Ogawa, Nader Sheibani, Maria T. Diaz-Meco, Jorge Moscat, Hiroshi Seno

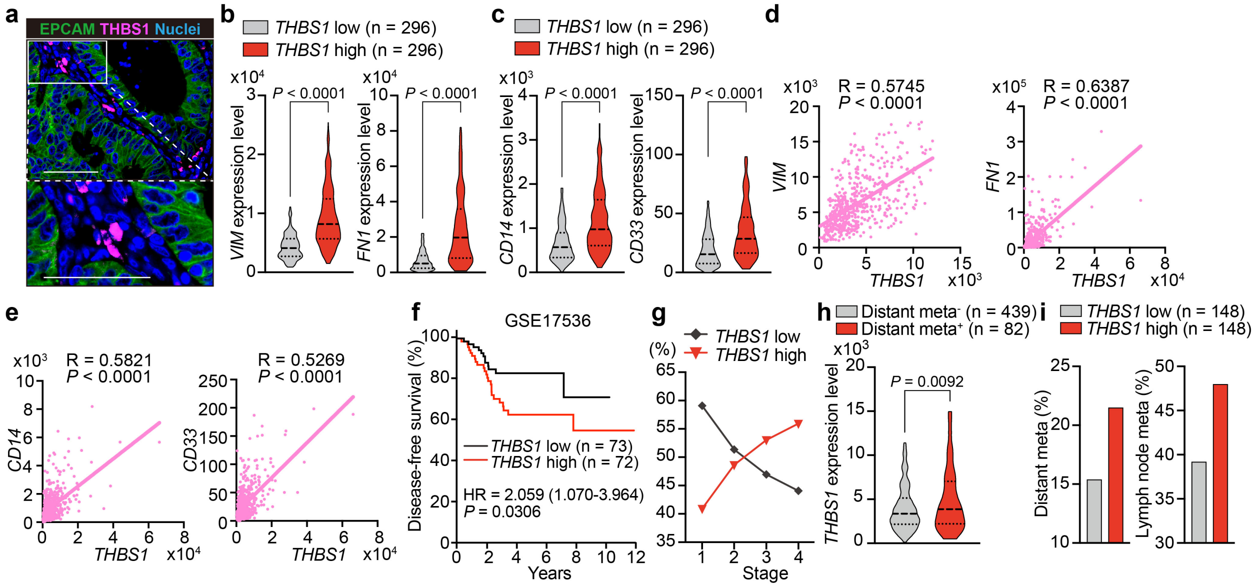

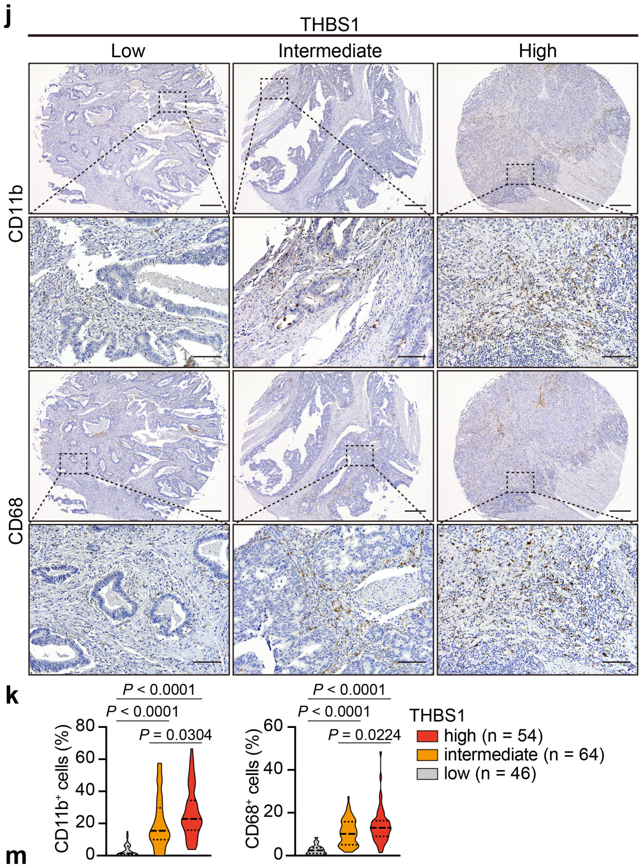

l

Varibles	All patients (n = 331)	THBS1 high (n = 107)	THBS1 intermediate (n = 122)	THBS1 low (n = 102)	*P* value
Age, years	67.2 (11.2)	66.9 (11.9)	67.4 (10.5)	67.4 (11.2)	0.983
Age ≧ 65	202 (60.8)	65 (60.7)	74 (60.7)	63 (61.8)	
Sex	169 (50.9)	43 (40.2)	62 (50.8)	63 (61.8)	0.008
Stage					0.002
1	3 (0.9)	3 (2.8)	0	0	
2	178 (53.6)	44 (41.1)	66 (54.1)	67 (65.7)	
3	151 (45.5)	60 (41.1)	56 (45.9)	35 (34.3)	
TNM classification T					0.385
T1	7 (2.1)	4 (3.7)	2 (1.6)	1 (0.98)	
T2	16 (4.8)	8 (7.5)	4 (3.3)	4 (3.9)	
T3	196 (59.0)	65 (60.7)	72 (59.0)	58 (56.9)	
T4	113 (34.0)	30 (28.0)	44 (36.1)	39 (38.2)	
TNM classification N					0.035
N0	181 (54.4)	47 (43.9)	66 (54.1)	67 (65.7)	
N1	107 (32.2)	41 (38.3)	44 (36.1)	22 (21.6)	
N2	37 (11.1)	15 (14.0)	11 (9.0)	11 (10.8)	
N3	7 (2.1)	4 (3.7)	1 (0.82)	2 (2.0)	
Primary location Proximal	100 (30.1)	36 (33.6)	37 (30.3)	27 (26.5)	0.528
Lymphatic invasion (n = 322)	232 (72.0)	82 (78.1)	78 (65.0)	72 (74.2)	0.078
Venous invasion	71 (21.4)	19 (17.8)	26 (21.3)	26 (25.5)	0.395
MSI-H (n = 301)	97 (29.2)	19 (19.6)	41 (36.0)	37 (41.6)	0.003
CMS4 (n = 118)	30 (25.4)	15 (36.6)	11 (22.0)	4 (14.8)	0.100
Cancer death	44 (13.3)	24 (22.4)	14 (11.5)	6 (5.9)	0.002
Recurrence	78 (23.5)	35 (32.7)	28 (23.0)	15 (14.7)	0.009
Mortality	70 (21.1)	30 (28.0)	28 (23.0)	12 (11.8)	0.013
Undifferentiated	24 (7.2)	13 (12.1)	8 (6.6)	3 (2.9)	0.035

Values are mean (standard deviation) or number (%).

m

Varibles	Recurrence		Lymph node met		Undifferentiated		MSS	
	OR (95%CI)	*P* value	OR (95%CI)	*P* value	OR (95%CI)	*P* value	OR (95%CI)	*P* value
THBS1		0.046		0.019		0.022		0.004
low	1 (reference)		1 (reference)		1 (reference)		1 (reference)	
intermediate	1.58 (0.76-3.29)		1.67 (0.95-2.96)		2.86 (0.69-11.8)		1.29 (0.72-2.30)	
high	2.48 (1.19-5.18)		2.32 (1.27-4.22)		5.72 (1.44-22.7)		2.94 (1.50-5.74)	

Supplementary Fig. 1 THBS1 expression positively correlated with mesenchyme- and myeloid-related genes and poor prognosis of CRC. a, Representative image of co-immunostaining for EPCAM and THBS1 in human CRC from three independent experiments with similar results. **b and c,** Transcript levels of mesenchyme- (**b**) and myeloid-related genes (**c**) in TCGA. **d and e,** Correlation of *THBS1* with indicated genes in TCGA (n = 592). **f,** Kaplan-Meier curve for disease-free survival in human CRC dataset (GSE17536). Hazard ratio with 95% confidence interval and *P* values, analyzed by Log-rank test, are shown. **g,** Proportion of stages 1 to 4 in *THBS1*-low (lower quartile) and *THBS1*-high CRC (upper quartile) in TCGA. **h,** Violin plot for *THBS1* expression in CRC with or without distant metastasis. **i,** Proportion of CRC with distant metastasis (left) or lymph node metastasis (right) in *THBS1*-low (lower quartile) and *THBS1*-high CRC (upper quartile) in TCGA. **j and k,** Immunostaining for CD11b and CD68 in CRC TMA samples (**j**) and proportion of positive cells (**k**). **l,** Basal characteristics and univariable analysis in CRC TMA samples using Pearson's chi-squared test. **m,** Summary of multivariable logistic regression analyses for indicated factors in column label. Detailed results of univariable and multivariable analyses for each factor are shown in Supplementary Tables 1–4. Scale bars, 25 μm (**a**), 200 μm (**j**, top), 50 μm (**j**, bottom). Mean ± SEM. *P* values were calculated by two-tailed Mann–Whitney test in (**b**,**c**), Pearson's correlation analyses (**d**,**e**), and two-tailed, unpaired Student's *t* test in (**h**,**k**). Source data are provided as a Source Data file.

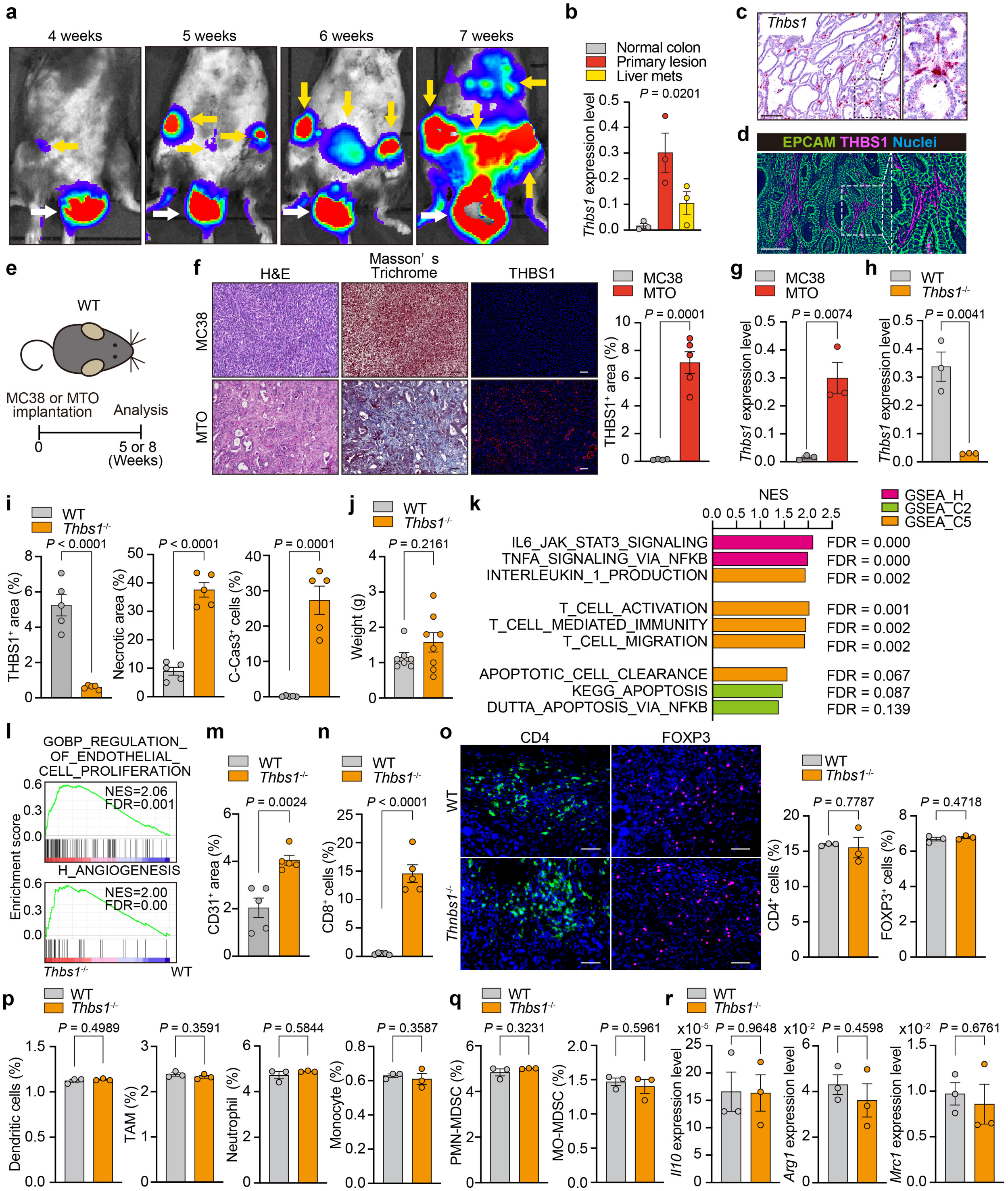
a
4 weeks
5 weeks
6 weeks
7 weeks
b
Normal colon
Primary lesion
Liver mets
P = 0.0201
Thbs1 expression level
c
Thbs1
d
EPCAM THBS1 Nuclei
e
WT
MC38 or MTO implantation
Analysis
0
5 or 8 (Weeks)
f
H&E
Masson' s Trichrome
THBS1
MC38
MTO
P = 0.0001
THBS1+ area (%)
g
MC38
MTO
P = 0.0074
Thbs1 expression level
h
WT
Thbs1-/-
P = 0.0041
Thbs1 expression level
i
WT
Thbs1-/-
P < 0.0001
THBS1+ area (%)
P < 0.0001
Necrotic area (%)
P = 0.0001
C-Cas3+ cells (%)
j
WT
Thbs1-/-
P = 0.2161
Weight (g)
k
NES
GSEA_H
GSEA_C2
GSEA_C5
IL6_JAK_STAT3_SIGNALING FDR = 0.000
TNFA_SIGNALING_VIA_NFKB FDR = 0.000
INTERLEUKIN_1_PRODUCTION FDR = 0.002
T_CELL_ACTIVATION FDR = 0.001
T_CELL_MEDIATED_IMMUNITY FDR = 0.002
T_CELL_MIGRATION FDR = 0.002
APOPTOTIC_CELL_CLEARANCE FDR = 0.067
KEGG_APOPTOSIS FDR = 0.087
DUTTA_APOPTOSIS_VIA_NFKB FDR = 0.139
l
GOBP_REGULATION_OF_ENDOTHELIAL_CELL_PROLIFERATION
NES=2.06
FDR=0.001
H_ANGIOGENESIS
NES=2.00
FDR=0.00
Enrichment score
Thbs1-/-
WT
m
WT
Thbs1-/-
P = 0.0024
CD31+ area (%)
n
WT
Thbs1-/-
P < 0.0001
CD8+ cells (%)
o
CD4
FOXP3
WT
Thnbs1-/-
WT
Thbs1-/-
P = 0.7787
CD4+ cells (%)
P = 0.4718
FOXP3+ cells (%)
p
WT
Thbs1-/-
P = 0.4989
Dendritic cells (%)
P = 0.3591
TAM (%)
P = 0.5844
Neutrophil (%)
P = 0.3587
Monocyte (%)
q
WT
Thbs1-/-
P = 0.3231
PMN-MDSC (%)
P = 0.5961
MO-MDSC (%)
r
WT
Thbs1-/-
x10-5 P = 0.9648
Il10 expression level
x10-2 P = 0.4598
Arg1 expression level
x10-2 P = 0.6761
Mrc1 expression level

Supplementary Fig. 2 THBS1 is highly expressed in the stroma of mesenchymal tumor and suppresses inflammation, T cell activity, and tumor cell apoptosis. a, Bioluminescence imaging of orthotopic implantation of MTO to WT mouse rectum. Arrows: primary lesions (white), distant metastases (yellow). **b,** qRT-PCR analysis of *Thbs1* expression in orthotopic MTO tumor-bearing mice (n = 3). **c,** Representative image of RNAscope for *Thbs1* in the orthotopic MTO tumors in WT mice from two independent experiments with similar results. **d,** Representative image of co-immunostaining for EPCAM and THBS1 in orthotopic WT tumors from three independent experiments with similar results. **e,** Schematic representation of orthotopic implantation of MC38 or MTO to WT mouse rectum. **f,** Staining in the orthotopic MC38 (n = 4) and MTO (n = 5) tumors, and quantification of THBS1 staining. **g,** qRT-PCR analysis in the orthotopic MC38 or MTO tumors (n = 3). **h,** qRT-PCR analysis in the orthotopic MTO tumors in WT and *Thbs1*$^{-/-}$ mice (n = 3). **i,** Quantification of Fig. 2**b** (n = 5). **j,** The weights of the orthotopic MTO tumors in WT (n = 7) and *Thbs1*$^{-/-}$ (n = 8) mice. **k and l,** GSEA on RNAseq data of orthotopic MTO tumors in WT or *Thbs1*$^{-/-}$ mice (n = 3). Normalized enrichment scores (NES) of indicated gene sets upregulated in *Thbs1*$^{-/-}$ mice (**k**) and enrichment score curves (**l**) of GSEA. **m and n,** Quantification (n = 5) of Fig. 2**d** (**m**) and Fig. 2**f** (**n**). **o,** Immunostaining and quantification (n = 3) in the orthotopic MTO tumors in WT or *Thbs1*$^{-/-}$ mice. **p and q**, FACS analyses of proportion of indicated cells in total cells in primary tumors in WT and *Thbs1*$^{-/-}$ mice (n = 3). Dendritic cells, CD45^{+}CD11c^{+}; TAM, CD11b^{+}F4/80^{+}; neutrophils, CD45^{+}CD11c^{-}CD11b^{+}Ly6C^{+}Ly6G^{+}F4/80^{-}; monocytes, CD45^{+}CD11c^{-}CD11b^{+}Ly6C^{+}Ly6G^{-}F4/80^{-}; PMN-MDSC, CD45^{+}CD11c^{-}CD11b^{+}Ly6C^{+}Ly6G^{+}; MO-MDSC, CD45^{+}CD11c^{-}CD11b^{+}Ly6C^{+}Ly6G^{-} cells. **r**, qRT-PCR analyses for indicated genes in the orthotopic MTO tumors in indicated mice (n = 3). Scale bars, 50 μm. Mean ± SEM. *P* values were calculated by one-way ANOVA in **b**, two-tailed, unpaired Student's *t* test in the rest**.** Source data are provided as a Source Data file.

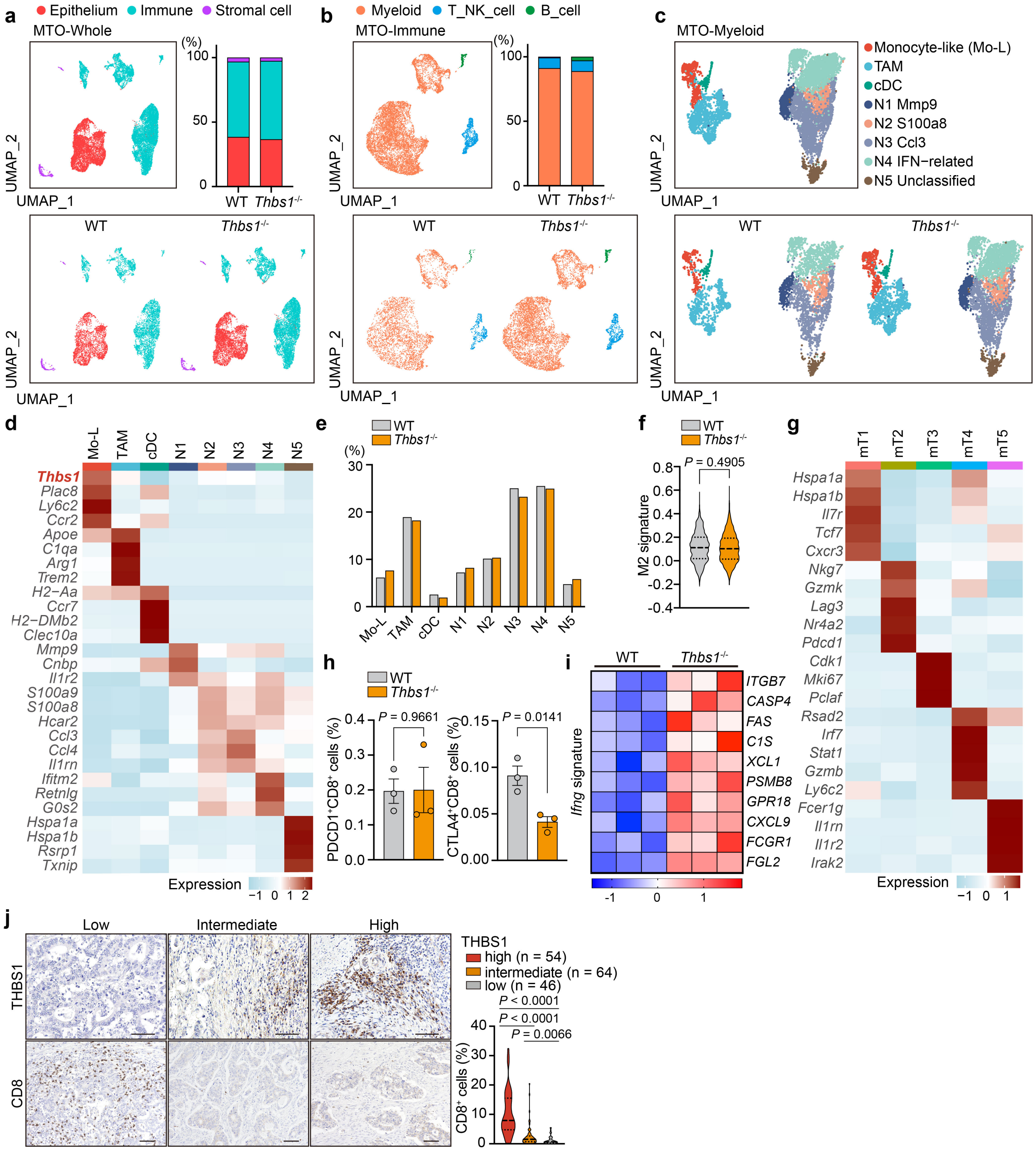
a
Epithelium
Immune
Stromal cell
MTO-Whole
(%)
100
50
0
WT
Thbs1-/-
UMAP_1
UMAP_2
b
Myeloid
T_NK_cell
B_cell
MTO-Immune
c
MTO-Myeloid
Monocyte-like (Mo-L)
TAM
cDC
N1 Mmp9
N2 S100a8
N3 Ccl3
N4 IFN−related
N5 Unclassified
d
Mo-L
TAM
cDC
N1
N2
N3
N4
N5
Thbs1
Plac8
Ly6c2
Ccr2
Apoe
C1qa
Arg1
Trem2
H2−Aa
Ccr7
H2−DMb2
Clec10a
Mmp9
Cnbp
Il1r2
S100a9
S100a8
Hcar2
Ccl3
Ccl4
Il1rn
Ifitm2
Retnlg
G0s2
Hspa1a
Hspa1b
Rsrp1
Txnip
Expression
−1 0 1 2
e
WT
Thbs1-/-
(%)
30
20
10
0
f
M2 signature
P = 0.4905
0.8
0.6
0.4
0.2
0.0
−0.2
−0.4
g
mT1
mT2
mT3
mT4
mT5
Hspa1a
Hspa1b
Il7r
Tcf7
Cxcr3
Nkg7
Gzmk
Lag3
Nr4a2
Pdcd1
Cdk1
Mki67
Pclaf
Rsad2
Irf7
Stat1
Gzmb
Ly6c2
Fcer1g
Il1rn
Il1r2
Irak2
Expression
−1 0 1
h
PDCD1+CD8+ cells (%)
P = 0.9661
CTLA4+CD8+ cells (%)
P = 0.0141
0.4
0.3
0.2
0.1
0.0
0.15
0.10
0.05
0.00
i
Ifng signature
ITGB7
CASP4
FAS
C1S
XCL1
PSMB8
GPR18
CXCL9
FCGR1
FGL2
-1
0
1
j
Low
Intermediate
High
THBS1
CD8
THBS1
high (n = 54)
intermediate (n = 64)
low (n = 46)
P < 0.0001
P < 0.0001
P = 0.0066
CD8+ cells (%)
40
30
20
10
0

Supplementary Fig. 3 Immune landscapes of orthotopic MTO tumors in WT or *Thbs1*$^{-/-}$ mice. a, UMAP plots for whole cells and proportion of each compartment in scRNA-seq of the orthotopic MTO tumors in WT and *Thbs1*$^{-/-}$ mice (n = 2 tumors from two distinct mice per group, analyzed cell numbers are indicated in **Methods** "**Single-cell RNA sequencing**"). **b,** UMAP plots and proportion of re-clustered immune subsets in (**a**). **c–e,** UMAP plots (**c**), heatmap for representative genes (**d**), and proportions (**e**) of re-clustered myeloid subsets in (**b**). **f,** Violin plot for M2-related macrophage signature in re-clustered myeloid subsets of WT and *Thbs1*$^{-/-}$ tumors in (**b**). **g,** Heatmap for representative genes of each cluster of CD8 T subset in Fig. 2**g**. **h,** FACS analyses of proportion of indicated cells in total cells in the orthotopic MTO tumors in WT or *Thbs1*$^{-/-}$ mice (n = 3). **i,** Heatmap of *Ifng* gene signature (Hallmark Collection of MSigDB) on the RNA-seq data from WT and *Thbs1*$^{-/-}$ tumors (n = 3). **j,** Immunostaining in human CRC with low-, intermediate- or high-intensity of THBS1 in TMA samples. Scale bars, 50 μm. Mean ± SEM. *P* values were calculated by two-tailed, unpaired Student's *t* test**.** Source data are provided as a Source Data file.

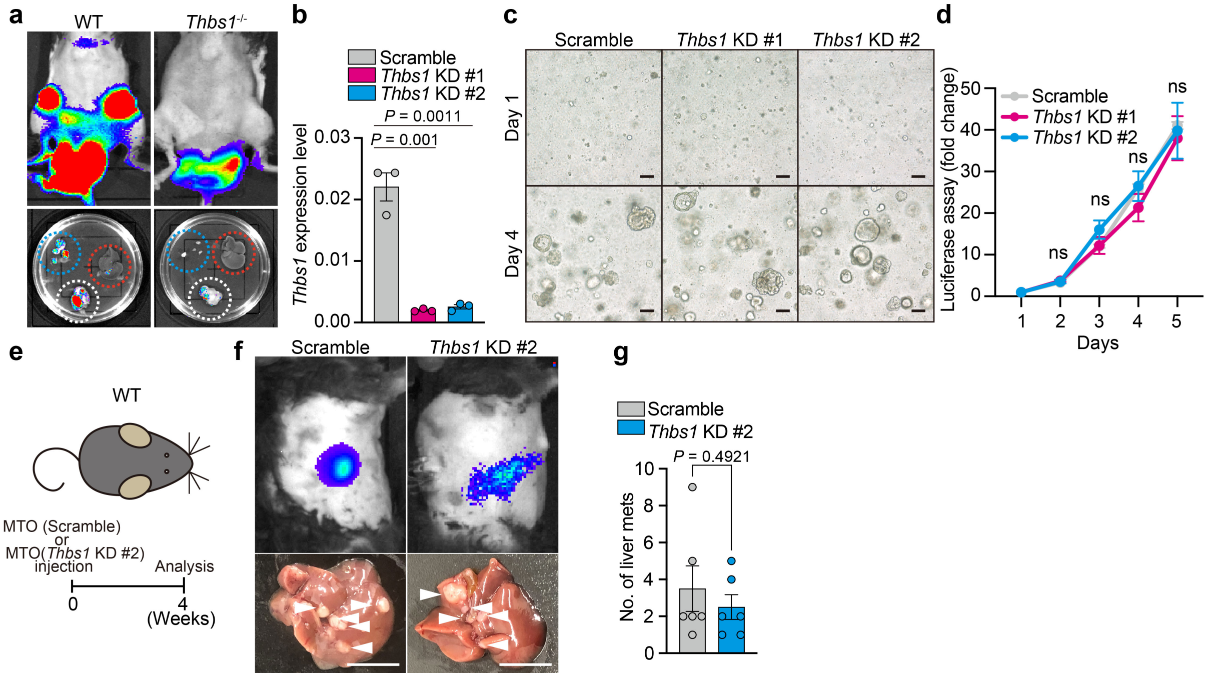

Supplementary Fig. 4 THBS1 loss in tumor epithelium does not affect progression of orthotopic MTO tumors. a, In vivo (top) and ex vivo (bottom) bioluminescence imaging of orthotopically MTO-inoculated WT or *Thbs1*$^{-/-}$ mice. Dash lines denote primary rectal tumor (white), liver (red), or lymph nodes (blue). **b,** qRT-PCR analysis in control (scramble) or *Thbs1*-knockdown (*Thbs1* KD) MTO (n = 3). **c and d,** MTO growth assay. Representative images of organoids (**c**), and growth curve (n = 3), measured by luminescence intensity at indicated time (**d**, relative value to day 1). **e,** Schematic representation of splenic injection of *Thbs1*-knockdown MTO to WT mice (n = 6). **f and g,** Bioluminescence imaging (**f**, top) and macroscopic images (**f**, bottom) of liver, and numbers of liver metastases (**g**) of (**e**)**.** White arrowheads denote liver metastases. Scale bars, 100 μm (**c**), 1 cm (**f**). Mean ± SEM. *P* values were calculated by two-tailed, unpaired Student's *t* test**.** Exact *P*-values are shown in the source data. Source data are provided as a Source Data file.

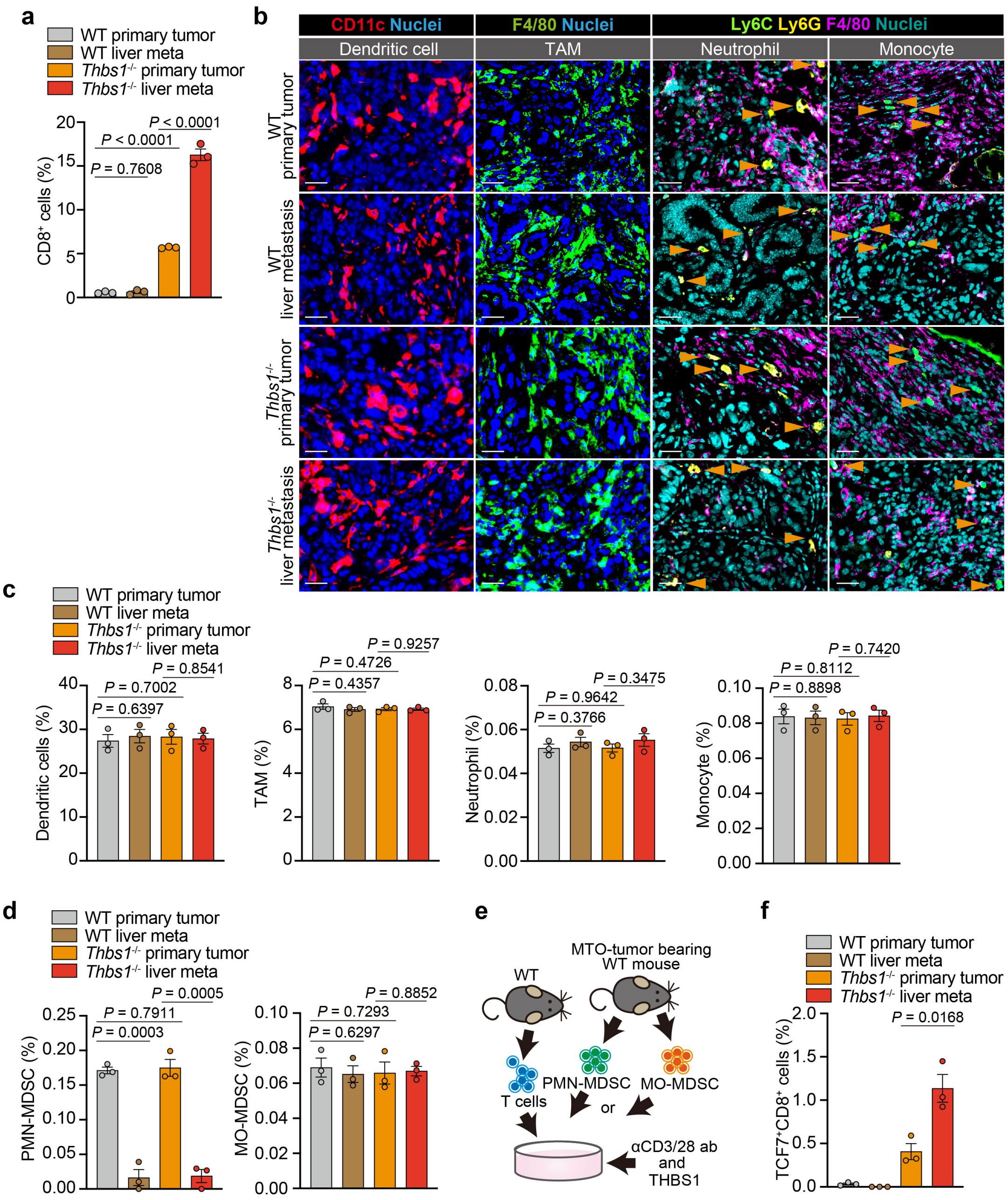
a
WT primary tumor
WT liver meta
Thbs1-/- primary tumor
Thbs1-/- liver meta
CD8+ cells (%)
P = 0.7608
P < 0.0001
P < 0.0001
b
CD11c Nuclei
F4/80 Nuclei
Ly6C Ly6G F4/80 Nuclei
Dendritic cell
TAM
Neutrophil
Monocyte
WT primary tumor
WT liver metastasis
Thbs1-/- primary tumor
Thbs1-/- liver metastasis
c
Dendritic cells (%)
P = 0.6397
P = 0.7002
P = 0.8541
TAM (%)
P = 0.4357
P = 0.4726
P = 0.9257
Neutrophil (%)
P = 0.3766
P = 0.9642
P = 0.3475
Monocyte (%)
P = 0.8898
P = 0.8112
P = 0.7420
d
PMN-MDSC (%)
P = 0.0003
P = 0.7911
P = 0.0005
MO-MDSC (%)
P = 0.6297
P = 0.7293
P = 0.8852
e
WT
MTO-tumor bearing WT mouse
T cells
PMN-MDSC
MO-MDSC
or
αCD3/28 ab and THBS1
f
TCF7+CD8+ cells (%)
P = 0.0168

Supplementary Fig. 5 Impact of THBS1 loss on the immune TME of primary and metastatic lesions in orthotopic MTO tumors. a, Quantification of Fig. 3**m** (n = 3). **b and c,** Immunostaining for CD11c or F4/80 and co-immunostaining (**b**) for Ly6C, Ly6G and F4/80 in primary tumors and metastatic liver tumors and quantification (**c**, n = 3) in orthotopically MTO-inoculated WT or *Thbs1*$^{-/-}$ mice. Orange arrows denote indicated cellular types. Scale bars, 50 μm (**b**). **d,** Quantification of Fig. 3q (n = 3). PMN-MDSC, Ly6C^{+}Ly6G^{+}; MO-MDSC, Ly6C^{+}Ly6G^{-} cells. **e,** Schematic representation of MDSC assay. **f,** Quantification of TCF7^{+}CD8^{+} cells in Fig. 3**s** (n = 3). Mean ± SEM. *P* values were calculated by two-tailed, unpaired Student's *t* test**.** Source data are provided as a Source Data file.

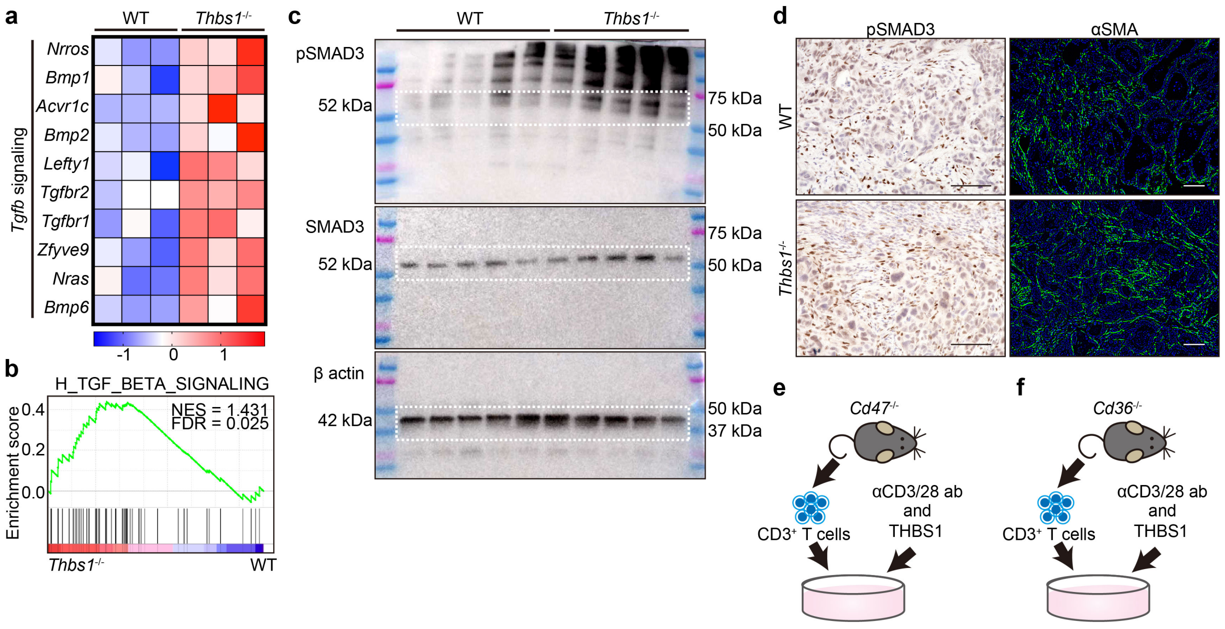

Supplementary Fig. 6 TGFβ signaling pathway in orthotopic MTO tumors in WT or *Thbs1*$^{-/-}$ mice. a and **b,** Heatmap of representative genes (**a**) and GSEA (**b**) of TGFβ signaling pathway signature (Hallmark Collection of MSigDB) on the RNA-seq data from the orthotopic MTO tumors in WT or *Thbs1*$^{-/-}$ mice (n = 3). **c,** Immunoblotting of phospho-SMAD3 (pSMAD3) and SMAD3 in the orthotopic MTO tumors in WT or *Thbs1*$^{-/-}$ mice (n = 5). Dash lines denote predicted molecular weight for each protein. **d,** Representative immunostaining of pSMAD3 and αSMA in the orthotopic MTO tumors in WT or *Thbs1*$^{-/-}$ mice from three independent experiments with similar results. Scale bars, 50 μm. **e and f,** Schematic representations of anti-CD3/CD28-mediated stimulation experiment of CD3^{+} T cells isolated from *Cd47*$^{-/-}$ (**e**) and *Cd36*$^{-/-}$ (**f**) mice. Source data are provided as a Source Data file.

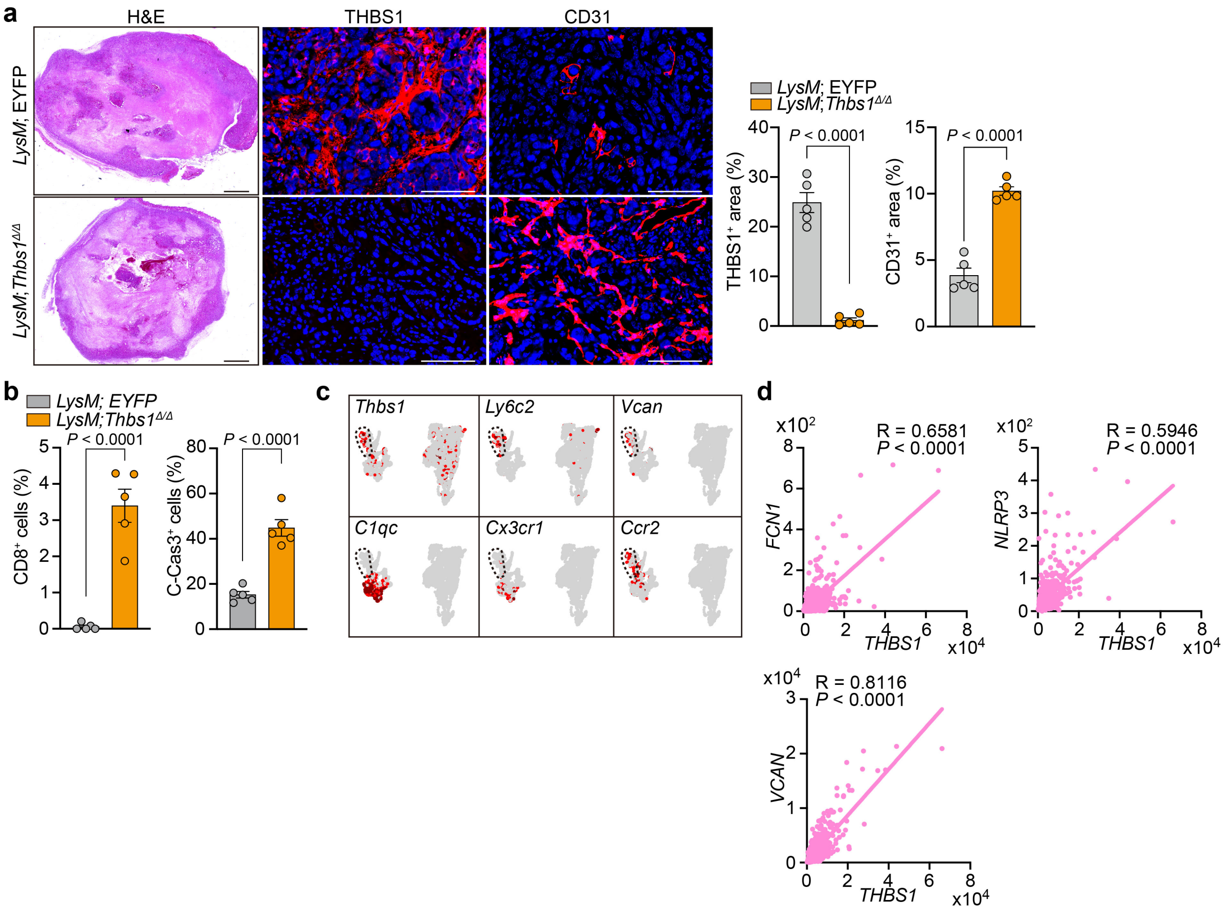

Supplementary Fig. 7 THBS1 is produced by monocyte/macrophage lineages in mesenchymal CRC. a, H&E and immunostaining in the orthotopic MTO tumors in *LysM;EYFP* or *LysM;Thbs1*$^{Δ/Δ}$ mice and quantification (n = 5). Scale bars, 200 μm (H&E), 50 μm (the rest). **b,** Quantification of (Fig. 5**g**, n = 5). **c,** Dot plots of expression of selected genes in scRNA-seq data of the orthotopic MTO tumors in Supplementary Fig. 3**c**. Dash lines denote monocyte-like cluster. **d,** Correlation of *THBS1* with *FCN1*, *NLRP3*, or *VCAN* in TCGA (n = 592). Mean ± SEM. *P* values were calculated by two-tailed, unpaired Student's *t* test (**a**,**b**) or Pearson's correlation analyses (**d**). Source data are provided as a Source Data file.

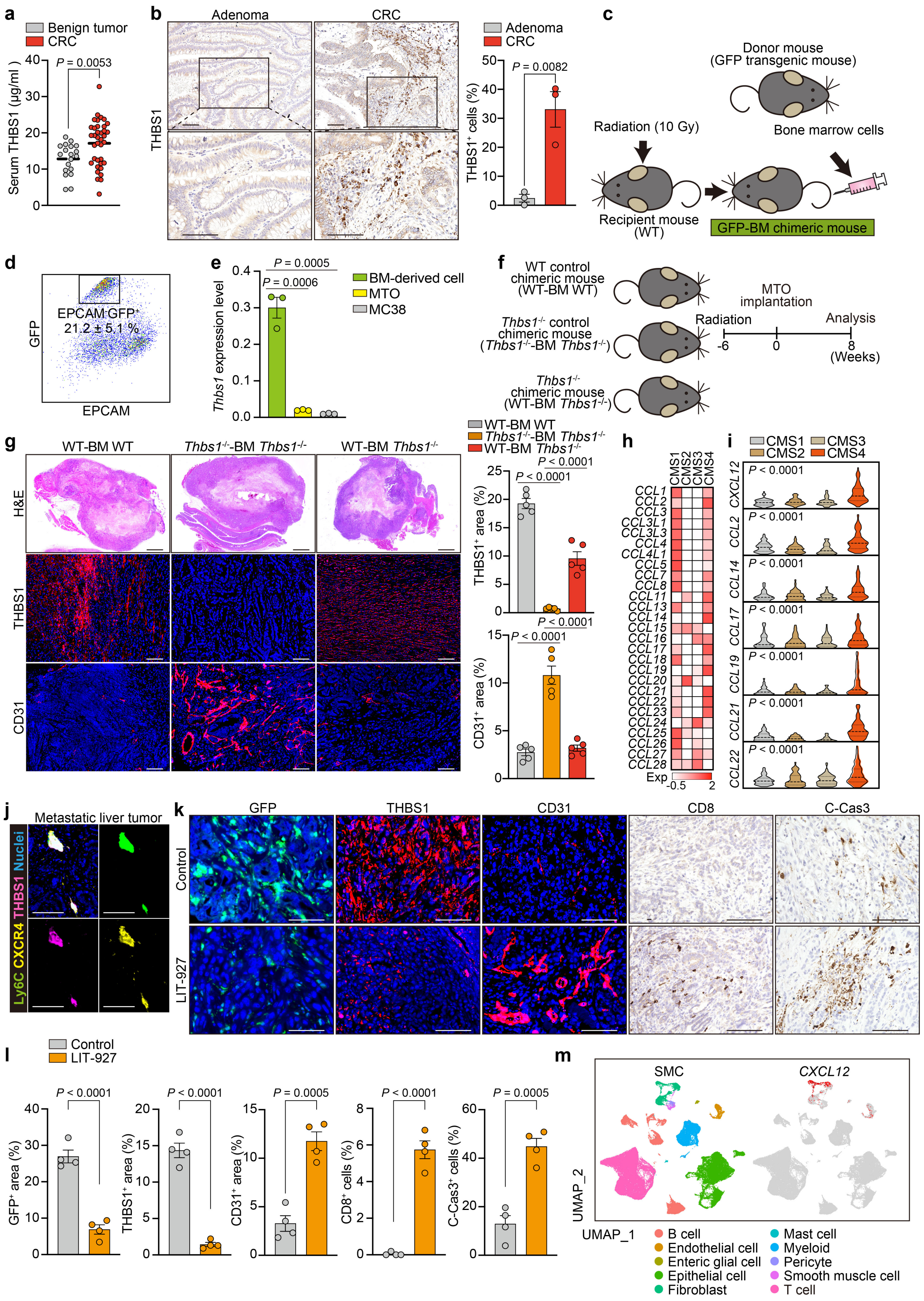

a
Benign tumor
CRC
P = 0.0053
Serum THBS1 (μg/ml)
b
Adenoma
CRC
THBS1
Adenoma
CRC
P = 0.0082
THBS1+ cells (%)
c
Donor mouse
(GFP transgenic mouse)
Radiation (10 Gy)
Bone marrow cells
Recipient mouse
(WT)
GFP-BM chimeric mouse
d
EPCAM-GFP+
21.2 ± 5.1 %
GFP
EPCAM
e
P = 0.0005
P = 0.0006
BM-derived cell
MTO
MC38
Thbs1 expression level
f
WT control
chimeric mouse
(WT-BM WT)
Thbs1-/- control
chimeric mouse
(Thbs1-/--BM Thbs1-/-)
Thbs1-/-
chimeric mouse
(WT-BM Thbs1-/-)
MTO
implantation
Radiation
Analysis
-6
0
8
(Weeks)
g
WT-BM WT
Thbs1-/--BM Thbs1-/-
WT-BM Thbs1-/-
H&E
THBS1
CD31
P < 0.0001
P < 0.0001
THBS1+ area (%)
P < 0.0001
P < 0.0001
CD31+ area (%)
h
CMS1
CMS2
CMS3
CMS4
CCL1
CCL2
CCL3
CCL3L1
CCL3L3
CCL4
CCL4L1
CCL5
CCL7
CCL8
CCL11
CCL13
CCL14
CCL15
CCL16
CCL17
CCL18
CCL19
CCL20
CCL21
CCL22
CCL23
CCL24
CCL25
CCL26
CCL27
CCL28
Exp
-0.5
2
i
CMS1
CMS3
CMS2
CMS4
CXCL12
CCL2
CCL14
CCL17
CCL19
CCL21
CCL22
P < 0.0001
j
Metastatic liver tumor
Ly6C CXCR4 THBS1 Nuclei
k
GFP
THBS1
CD31
CD8
C-Cas3
Control
LIT-927
l
Control
LIT-927
P < 0.0001
P < 0.0001
P = 0.0005
P < 0.0001
P = 0.0005
GFP+ area (%)
THBS1+ area (%)
CD31+ area (%)
CD8+ cells (%)
C-Cas3+ cells (%)
m
SMC
CXCL12
UMAP_2
UMAP_1
B cell
Endothelial cell
Enteric glial cell
Epithelial cell
Fibroblast
Mast cell
Myeloid
Pericyte
Smooth muscle cell
T cell

Supplementary Fig. 8 THBS1-expressing monocyte-like cells are recruited to primary tumor site via CXCL12/CXCR4 signaling. a, Serum THBS1 levels in patients with CRC (n = 37) or benign tumors (n: adenoma = 15, sessile serrated lesion = 4). **b,** Immunostaining of THBS1 in human adenoma and CRC and quantification (n = 3). Scale bars, 50 μm. **c**, Schematic representation of generation of GFP-BM chimeric mice. **d**, Representative FACS analyses of orthotopic MTO tumors in GFP-BM chimeric mice (n = 3). **e**, qRT-PCR analysis for *Thbs1* in BM-derived ($EPCMA^{-}GFP^{+}$) cells in GFP-BM chimeric mice compared to MTO and MC38 cells (n = 3). **f,** Schematic representation of orthotopic implantation of MTO to the indicated BM-chimeric mice. **g,** H&E and immunostaining in the orthotopic MTO tumors of indicated BM-chimeric mice and quantification (n = 5). Scale bars, 200 μm (H&E), 50 μm (the rest). **h**, Heatmap for transcript expression of CC-chemokines in CRC from TCGA, stratified by CMS subtypes. **i,** Transcript levels of indicated genes in TCGA stratified by CMS classification (n: CMS1 = 85, CNS2 = 132, CMS3 = 78, CMS4 = 184). **j,** Representative co-immunostaining for Ly6C, CXCR4 and THBS1 in liver metastasis in orthotopically MTO-inoculated WT mice from three independent experiments with similar results. Scale bars 50 μm. **k and l,** Immunostaining (**k**) and quantification (**l**; n = 5) in the orthotopic MTO tumors in GFP-BM chimeric mice with or without administration of LIT-927. Scale bars, 50 μm. **m,** UMAP plot for whole cells showing cellular subsets and *CXCL12*-expressing cells of human CRC scRNA-seq data (SMC). Mean ± SEM. *P* values were calculated by two-tailed Mann–Whitney test in (**a**), two-tailed unpaired Student's *t* test in (**b**,**e**,**g**,**l**), or one-way ANOVA in (**i**). Source data are provided as a Source Data file.

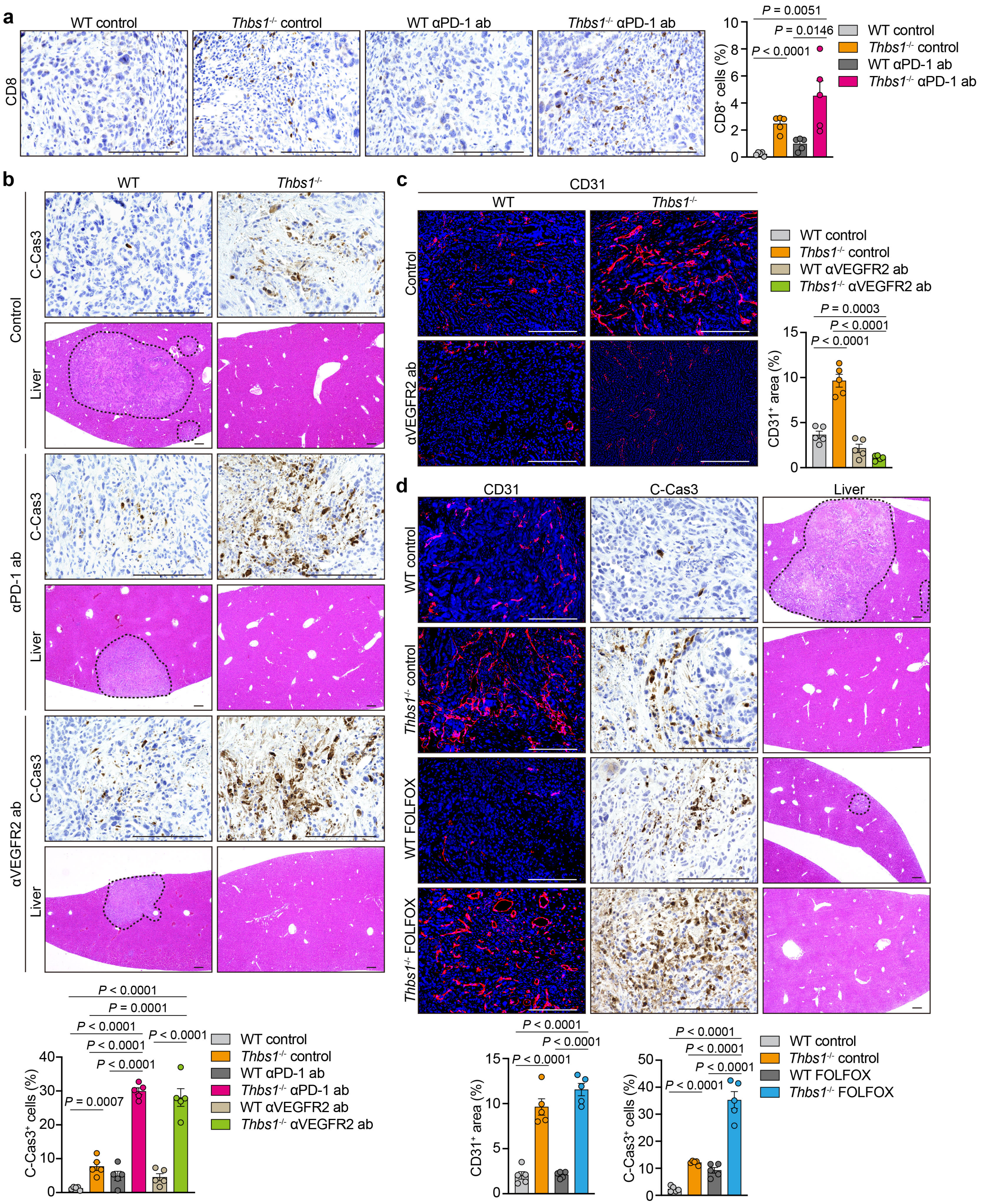

a
WT control
Thbs1-/- control
WT αPD-1 ab
Thbs1-/- αPD-1 ab
CD8
P = 0.0051
P = 0.0146
P < 0.0001
CD8+ cells (%)
WT control
Thbs1-/- control
WT αPD-1 ab
Thbs1-/- αPD-1 ab
b
WT
Thbs1-/-
Control
C-Cas3
Liver
αPD-1 ab
αVEGFR2 ab
P < 0.0001
P = 0.0001
P < 0.0001
P < 0.0001
P < 0.0001
P < 0.0001
P = 0.0007
C-Cas3+ cells (%)
WT control
Thbs1-/- control
WT αPD-1 ab
Thbs1-/- αPD-1 ab
WT αVEGFR2 ab
Thbs1-/- αVEGFR2 ab
c
CD31
WT
Thbs1-/-
Control
αVEGFR2 ab
WT control
Thbs1-/- control
WT αVEGFR2 ab
Thbs1-/- αVEGFR2 ab
P = 0.0003
P < 0.0001
P < 0.0001
CD31+ area (%)
d
CD31
C-Cas3
Liver
WT control
Thbs1-/- control
WT FOLFOX
Thbs1-/- FOLFOX
P < 0.0001
P < 0.0001
P < 0.0001
CD31+ area (%)
P < 0.0001
P < 0.0001
P < 0.0001
P < 0.0001
C-Cas3+ cells (%)
WT control
Thbs1-/- control
WT FOLFOX
Thbs1-/- FOLFOX

Supplementary Fig. 9 Loss of THBS1 partially improved response of aggressive CRC to current treatments a, Immunostaining for CD8 in the primary lesions in MTO-bearing WT or *Thbs1*$^{-/-}$ mice with or without administration of anti-PD-1 antibody (αPD-1 ab), and quantification (n = 5). **b,** Immunostaining for C-Cas3 in the primary lesions and H&E staining of liver in MTO-bearing WT or *Thbs1*$^{-/-}$ mice with or without administration of αPD-1 ab or anti-VEGFR2 antibody (αVEGFR2 ab), and quantification (n = 5). **c,** Immunostaining for CD31 in the primary lesions in MTO-bearing WT or *Thbs1*$^{-/-}$ mice with or without administration of αVEGFR2 ab, and quantification (n = 5). **d**, Immunostaining for CD31 and C-Cas3 in the primary lesions and H&E staining of liver in MTO-bearing WT or *Thbs1*$^{-/-}$ mice with or without administration of FOLFOX, and quantification (n = 5). Dash lines denote liver metastases. Mean ± SEM. *P* values were calculated by two-tailed, unpaired Student's *t* test. Scale bars, 100 μm. Source data are provided as a Source Data file.

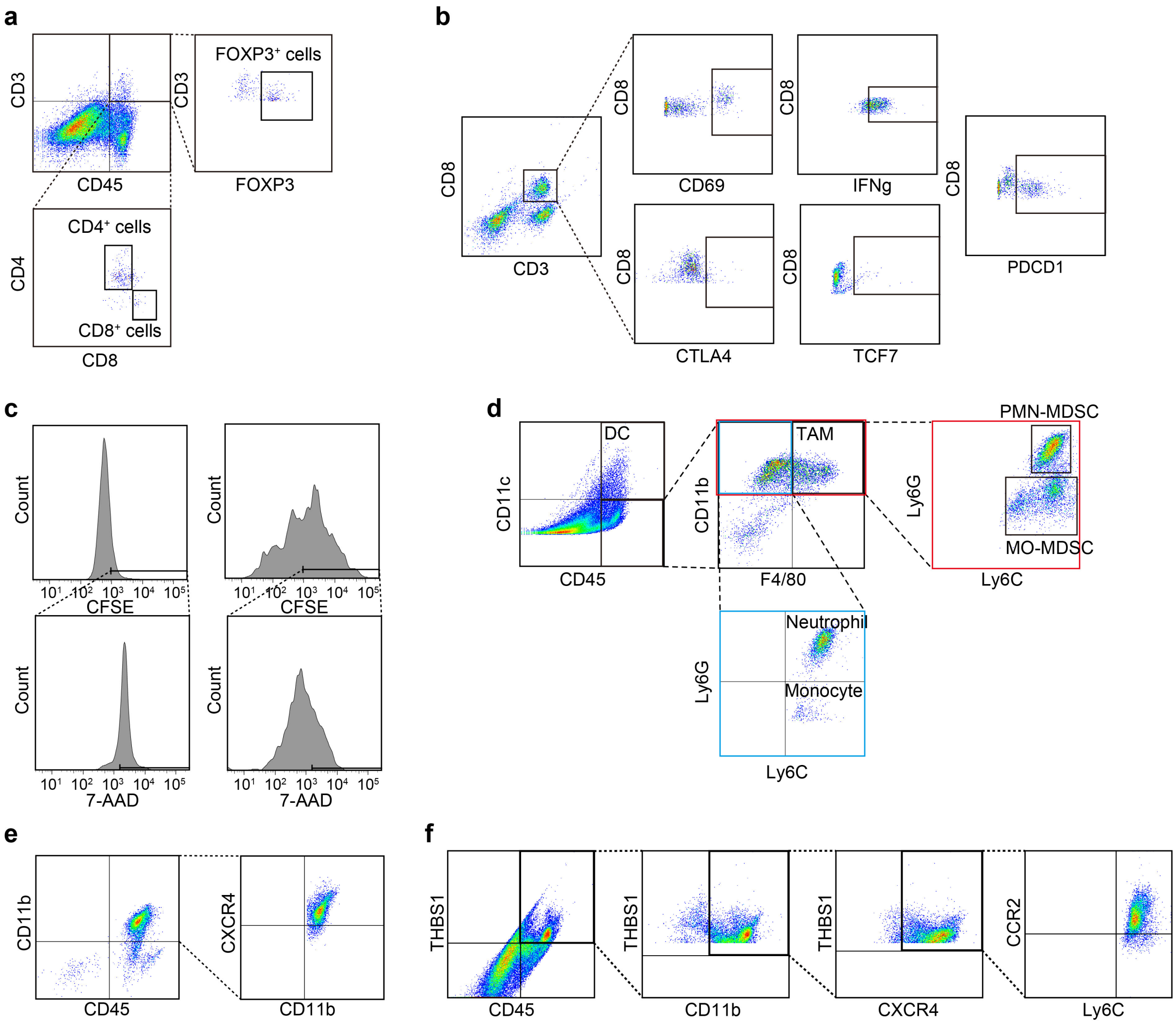
a
CD3
CD45
FOXP3⁺ cells
CD3
FOXP3
CD4⁺ cells
CD4
CD8⁺ cells
CD8
b
CD8
CD3
CD69
IFNg
CTLA4
TCF7
PDCD1
c
Count
CFSE
7-AAD
d
DC
CD11c
CD45
TAM
CD11b
F4/80
PMN-MDSC
Ly6G
MO-MDSC
Ly6C
Neutrophil
Monocyte
e
CD11b
CD45
CXCR4
f
THBS1
CCR2
Ly6C

Supplementary Fig. 10 FACS gating strategies a, Gating strategy for $CD4^+$ T, $CD8^+$ T, and $FOXP^+$ T cells from orthotopic MTO tumors, related with Fig. 2e and 3n. T cells were collected from dissociated tumor as $CD45^+CD3^+$ cells. $CD4^+$ T, $CD8^+$ T, or $FOXP3^+$ T cells were sorted from $CD45^+CD3^+$ cells. **b,** Gating strategies for active or dysfunctional $CD8^+$ T cells, related with Fig. 2n, 3r, 4h, and 4l and Supplementary Fig. 3h. Active $CD8^+$ T cells were collected as $CD3^+CD8^+CD69^+$ or $CD3^+CD8^+IFNg^+$ cells. Dysfunctional $CD8^+$ T cells were collected as $CD3^+CD8^+CTLA4^+$ or $Cd3^+CD8^+PDCD1^+$ cells. Stem-like $CD8^+$ T cells were collected as $CD3^+CD8^+TCF7^+$ cells. **c,** Gating strategies for cytotoxicity assay related with Fig. 2q. Dead cells were measured with 7-AAD in MTO, pre-stained with CFSE. **d,** Gating strategies for myeloid cells in orthotopic MTO tumors, related with Fig. 3o and 3p and Supplementary Fig. 2p and 2q. Dendritic cells, TAMs, neutrophils, monocytes, PMN-MDSC, and MO-MDSC were sorted as $CD45^+CD11c^+$, $CD45^+CD11c^-CD11b^+F4/80^+$, $CD45^+CD11c^-CD11b^+F4/80^-Ly6G^+Ly6C^+$, $CD45^+CD11c^-CD11b^+F4/80^-Ly6G^-Ly6C^+$, $CD45^+CD11c^-CD11b^+Ly6G^+Ly6C^+$, and $CD45^+CD11c^-CD11b^+Ly6G^-Ly6C^+$, respectively. **e,** Strategies for sorting $CD45^+CD11b^+CXCR4^+$ cells in BM or peripheral blood cells, related with Fig. 6a–c, 6o. **f,** Gating strategy for sorting $THBS1^+$ cells in orthotopic MTO tumors, related with Fig. 6p.

Supplementary Table 1 Univariable and multivariable logistic regression analysis for recurrence

Variables	Univariable analysis, odds ratio (95%CI)	*P* value	Multivariable analysis, odds ratio (95%CI)	*P* value
**THBS1**		0.008		0.046
**Low (reference)**	1		1	
**Intermediate**	1.73 (0.87-3.45)		1.58 (0.76-3.29)	
**High**	2.82 (1.43-5.57)		2.48 (1.19-5.18)	
**T**		0.064		0.097
**T1 (reference)**	1		1	
**T2**	0.36 (0.04-3.26)		0.34 (0.03-3.40)	
**T3**	0.61 (0.11-3.24)		0.98 (0.17-5.84)	
**T4**	1.17 (0.22-6.31)		1.64 (0.27-9.86)	
**N**		< 0.0001		0.564
**N0 (reference)**	1		1	
**N1**	3.46 (1.91-6.27)		1 (1)	
**N2**	4.65 (2.11-10.2)		1.28 (0.56-2.91)	
**N3**	9.10 (1.91-43.3)		2.25 (0.45-11.3)	
**Stage**		<0.0001		0.0009
**1 (reference)**	1		1	
**2**	598053.8 (-)		442015.7	
**3**	2294164.7 (-)		1378820.7	
**Histology type**		0.030		0.649
**Undifferentiated**	2.51 (1.07-5.90)		1.26 (0.47-3.35)	

Univariable analysis was performed using Pearson’s chi-squared test (left). Tow-sided and unadjusted multivariable logistic regression analysis for the factors associated with recurrence was performed (right).

Supplementary Table 2 Univariable and multivariable logistic regression analysis for lymph mode metastasis positive

Variables	Univariable analysis, odds ratio (95%CI)	*P* value	Multivariable analysis, odds ratio (95%CI)	*P* value
**THBS1**		0.007		0.019
**Low (reference)**	1		1	
**Intermediate**	1.62 (0.94-2.79)		1.67 (0.95-2.96)	
**High**	2.44 (1.40-4.28)		2.32 (1.27-4.22)	
**T**		< 0.0001		0 < 0.0001
**T1 (reference)**	1		1	
**T2**	1.17 (0.09-15.5)		1.23 (0.09-16.6)	
**T3**	0.09 (0.01-0.79)		0.10 (0.01-0.86)	
**T4**	0.20 (0.02-1.68)		0.21 (0.02-1.85)	
**Histology type**		0.010		0.056
**Undifferentiated**	3.14 (1.26-7.78)		2.45 (0.95-6.32)	

Univariable analysis was performed using Pearson's chi-squared test (left). Tow-sided and unadjusted multivariable logistic regression analysis for the factors associated with lymph node metastasis was performed (right).

Supplementary Table 3 Univariable and multivariable logistic regression analysis for undifferentiated histology

Variables	Univariable analysis, odds ratio (95%CI)	*P* value	Multivariable analysis, odds ratio (95%CI)	*P* value
**THBS1**		0.032		0.022
**Low (reference)**	1		1	
**Intermediate**	2.32 (0.60-8.97)		2.86 (0.69-11.8)	
**High**	4.56 (1.26-16.5)		5.72 (1.44-22.7)	
**T**		0.069		0.058
**T1 (reference)**	1		1	
**T2**	266957.4 (-)		1678320.7 (-)	
**T3**	193759.4 (-)		1361536.4 (-)	
**T4**	566273.2 (-)		4479693.3 (-)	
**N**		0.001		0.003
**N0 (reference)**	1		1	
**N1**	1.47 (0.48-4.49)		1 (-)	
**N2**	7.94 (2.74-23.1)		6.58 (2.03-21.4)	
**N3**	9.89 (1.63-60.1)		7.29 (1.04-51.2)	
**Stage**		0.031		0.087
**1 (reference)**	1		1	
**2**	164885.4 (-)		1181578.8 (-)	
**3**	508015.9 (-)		1122911.6 (-)	

Univariable analysis was performed using Pearson's chi-squared test (left). Tow-sided and unadjusted multivariable logistic regression analysis for the factors associated with undifferentiated histology was performed (right).

Supplementary Table 4 Univariable and multivariable logistic regression analysis for MSS status

Variables	Univariable analysis, odds ratio (95%CI)	*P* value	Multivariable analysis, odds ratio (95%CI)	*P* value
**THBS1**		0.003		0.004
**Low (reference)**	1		1	
**Intermediate**	1.27 (0.72-2.24)		1.29 (0.72-2.30)	
**High**	2.92 (1.52-5.56)		2.94 (1.50-5.74)	
**T**		0.020		0.082
**T1 (reference)**	1		1	
**T2**	1.42×10^{-6} (-)		6.16×10^{-7} (-)	
**T3**	5.75×10^{-7} (-)		2.58×10^{-7} (-)	
**T4**	3.75×10^{-7} (-)		1.85×10^{-7} (-)	
**Primary tumor location**		0.042		0.070
**Proximal**	0.59 (0.35-0.98)		0.61 (0.36-1.04)	

MSS: microsatellite stable.

Univariable analysis was performed using Pearson's chi-squared test (left). Tow-sided and unadjusted multivariable logistic regression analysis for the factors associated with MSS was performed (right).

Supplementary Table 5 Mouse qRT-PCR primers

Gene	Forward	Reverse
Thbs1	GAAGCAACAAGTGGTGTCAGT	ACAGTCTATGTAGAGTTGAGCCC
Ifng	ATGAACGCTACACACTGCATC	CCATCCTTTTGCCAGTTCCTC
Ctla4	GTACCTCTGCAAGGTGGAACTC	CCAAAGGAGGAAGTCAGAATCCG
Cd11b	ATGGACGCTGATGGCAATACC	TCCCCATTCACGTCTCCCA
Cxcl12	TGCATCAGTGACGGTAAACCA	TTCTTCAGCCGTGCAACAATC
Fcn1	CACCATCCATCTTCCTGACTGC	ATAGGAGTCCCAGTCTCGGAAG
Nlrp3	ATTACCCGCCCGAGAAAGG	CATGAGTGTGGCTAGATCCAAG
Ccl2	TTAAAAACCTGGATCGGAACCAA	GCATTAGCTTCAGATTTACGGGT
18s	GTAACCCGTTGAACCCATT	CCATCCAATCGGTAGTAGCG
IL10	GCTCTTACTGACTGGCATGAG	CGCAGCTCTAGGAGCATGTG
Vcan	TTTTACCCGAGTTACCAGACTCA	GGAGTAGTTGTTACATCCGTTGC
Arg1	TGGCTTGCGAGACGTAGAC	GCTCAGGTGAATCGGCCTTTT
Mrc1	GCTGAATCCCAGAAATTCCGC	ATCACAGGCATACAGGGTGAC

Supplementary Table 6 Representative genes in indicated signatures

Signature	Genes
**CMS4_UP** [1]	*MSRB3, FERMT2, EFEMP2, SPOCK1, DDR2, TAGLN, CCDC80, GLI3, TNS1, STON1, PTRF, SFRP2, MGP, GAS1, BNC2, SLIT2, DPYSL3, AEBP1, PCDH7, MAP1B, CRYAB, PRRX1, FBN1, MXRA8, PTGIS, ZFPM2, MLLT11, MYL9*
**CMS_DOWN** [1]	*SEPT1, RMI1, ASF1B, STIL, CCNA2, HMGB2, CDC45, KIF18A, UNG, WHSC1, KIF18B, HK2, RBM47, DONSON, PLK4, CCDC134, EIF4E, RHPN2, FAM83F, FANCD2, HOOK1, CDCA2, EZH2, GMCL1, CENPA, ORC1, SPAG5, TMEM54*
**Stem-like signature** [2]	*Tcf7, Xcl, Cxcr5, Ltb, Pdcd4, Irf3, Slamf6, Cd200*
**Dysfunction signature** [3]	*Ccl3, Cxcl13, Klr3dl1, Klr3dl2, Ifng, Cd7, Cd27, Akap5, Havcr2, Tnfrsf1b, Tnfrsf9, Lyst, Ptms, Tns3, Gbp2b, Entpd1, Pdcd1, Phlda1, Csf1, Rdh10, Ctla4, Dgkh, Snap47, Itgae, Il2rb*
**M2 macrophage-related signature** [4]	*Ccl4, Ccl13, Ccl20, Ccl22, Ccl276, Clec7a, Ctsa, Ctsb, Ctsc, Ctsd, Fn1, Il4ra, Irf4, Lyve1, Mmp9, Mmp14, Mmp19, Msr1, Tgfb1, Tgfb2, Tgfb3, Tnfsf8, Tbfsf12, Vegfa, Vegfb, Vegfc*

Reference

1. Tauriello, D. V. F. et al. TGFβ drives immune evasion in genetically reconstituted colon cancer metastasis. Nature 554, 538-543 (2018).
2. Connolly, K. A. et al. A reservoir of stem-like CD8. Sci Immunol 6, eabg7836 (2021).
3. Li, H. et al. Dysfunctional CD8 T Cells Form a Proliferative, Dynamically Regulated Compartment within Human Melanoma. Cell 176, 775-789.e718 (2019).
4. Azizi, E. et al. Single-Cell Map of Diverse Immune Phenotypes in the Breast Tumor Microenvironment. Cell 174, 1293-1308.e1236 (2018).
