# Supplementary material for: THBS1-producing tumor-infiltrating monocyte-like cells contribute to immunosuppression and metastasis in colorectal cancer
Source: Nat Commun. 2023 Sep 25;14:5534. doi: 10.1038/s41467-023-41095-y (PMC10520015; doi:10.1038/s41467-023-41095-y)
Supplement: Supplementary file 3 — Reporting Summary [file 41467_2023_41095_MOESM3_ESM.pdf]

nature portfolio

Corresponding author(s): Yuki Nakanishi

Last updated by author(s): Aug 8, 2023

Reporting Summary

Nature Portfolio wishes to improve the reproducibility of the work that we publish. This form provides structure for consistency and transparency in reporting. For further information on Nature Portfolio policies, see our Editorial Policies and the Editorial Policy Checklist.

Statistics

For all statistical analyses, confirm that the following items are present in the figure legend, table legend, main text, or Methods section.

n/a	Confirmed	
☐	☒	The exact sample size (*n*) for each experimental group/condition, given as a discrete number and unit of measurement
☐	☒	A statement on whether measurements were taken from distinct samples or whether the same sample was measured repeatedly
☐	☒	The statistical test(s) used AND whether they are one- or two-sided<br>*Only common tests should be described solely by name; describe more complex techniques in the Methods section.*
☐	☒	A description of all covariates tested
☐	☒	A description of any assumptions or corrections, such as tests of normality and adjustment for multiple comparisons
☐	☒	A full description of the statistical parameters including central tendency (e.g. means) or other basic estimates (e.g. regression coefficient) AND variation (e.g. standard deviation) or associated estimates of uncertainty (e.g. confidence intervals)
☐	☒	For null hypothesis testing, the test statistic (e.g. *F*, *t*, *r*) with confidence intervals, effect sizes, degrees of freedom and *P* value noted<br>*Give P values as exact values whenever suitable.*
☒	☐	For Bayesian analysis, information on the choice of priors and Markov chain Monte Carlo settings
☒	☐	For hierarchical and complex designs, identification of the appropriate level for tests and full reporting of outcomes
☐	☒	Estimates of effect sizes (e.g. Cohen's *d*, Pearson's *r*), indicating how they were calculated

Our web collection on statistics for biologists contains articles on many of the points above.

Software and code

Policy information about availability of computer code

Data collection	RNA-seq data generated in this study was collected by Macrogen, Inc on the Novaseq6000 platform. Data for TCGA-COREAD was accessed through cBioportal (http://www.cbioportal.org/index.do). Single cell RNA-seq data generated in this study was collected by a 10X Chromium Controller (10X Genomics), a DNBSEQ-G400RS (MGI). FACS data was collected by FACSaria Ⅱ (BD biosciences). qRT-PCR alaysis was performed on a LightCycler 96 (Roche Diagnostics). Immunoblotting images were collected by Amersham Imager 600 (GE Healthcare). On elisa analysis, colorimetry was performed on a maico plate reader Sunrise (TECAN). In vitro luciferase assay, luminescence was measured by SpectraMax iD5 (Molecular Devices). In vivo luciferase assay, luminescence was measured by IVIS Lumina Ⅱ (PerkinElmer). Histological and Immunostaining images were collected by BZ-X710 (KEYENCE). Cell images were collected by IX73 (Olympus).
Data analysis	For RNA seq analysis, adaptors and low-quality bases were trimmed from the reads using Trimmomatic (version 0.39) with default parameters, and reads were mapped to the Mus musculus reference genome build mm10 using STAR (version 2.7.3a) and counted by RSEM (version v1.3.1). Read count data were normalized using the iDEGES/edgeR method. Normalized count data were used for gene set enrichment analysis (GSEA) and differentially expressed genes were determined using TCC (Tag Count Comparison) with a false discovery rate cutoff value < 0.05. GSEA was performed using GSEA software (http://www.gsea-msigdb.org/gsea/index.jsp). For single-cell RNA seq analysis, the Cell Ranger version 6.1.1 software suite from 10X Genomics was used to process, align and summarize unique molecular identifier (UMI) counts. Additional data processing and analysis was performed using Seurat version 4.1.1 in R version 4.2.3. Cells expressing fewer than 200 genes and cells expressing greater than 20% mitochondrial related genes were removed. CRC samples were stratified according to CMS subtypes using CMScaller package (Peter W. Eide et al. Sci Rep 2017). FACS analysis was performed by Flowjo v.10. Quantification of images were performed on ImageJ/Fiji v.2.3.0/1.53f and Qupath v.0.3.2. Statical analysis were performed on Prism v.9.

For manuscripts utilizing custom algorithms or software that are central to the research but not yet described in published literature, software must be made available to editors and reviewers. We strongly encourage code deposition in a community repository (e.g. GitHub). See the Nature Portfolio guidelines for submitting code & software for further information.

Data

Policy information about availability of data

All manuscripts must include a data availability statement. This statement should provide the following information, where applicable:

- Accession codes, unique identifiers, or web links for publicly available datasets
- A description of any restrictions on data availability
- For clinical datasets or third party data, please ensure that the statement adheres to our policy

The data generated in this study are available in the Genomic Expression Archive (GEA) database under the accession numbers E-GEAD-561 for bulk RNA-seq [https://ddbj.nig.ac.jp/public/ddbj_database/gea/experiment/E-GEAD-000/E-GEAD-561/] and E-GEAD-562 for scRNA-seq data [https://ddbj.nig.ac.jp/public/ddbj_database/gea/experiment/E-GEAD-000/E-GEAD-562/]. Data for TCGA-COREAD was accessed through cBioportal (https://www.cbioportal.org). Raw gene expression data of CRC patient dataset (GSE35602 [https://www.ncbi.nlm.nih.gov/geo/query/acc.cgi?acc=GSE35602], GSE14333 [https://www.ncbi.nlm.nih.gov/geo/query/acc.cgi?acc=GSE14333] and GSE17536 [https://www.ncbi.nlm.nih.gov/geo/query/acc.cgi?acc=GSE17536]) were directly accessed through the GEO website (NCBI). Raw gene expression (count) data and metadata of single cell transcriptomic dataset of human CRCs (SMC) were directly accessed through the GEO website (GSE132465 [https://www.ncbi.nlm.nih.gov/geo/query/acc.cgi?acc=GSE132465]), and converted into Seurat object. Published single cell transcriptome data of orthotopic MTO tumor was downloaded from GEO website (GSE154863 [https://www.ncbi.nlm.nih.gov/geo/query/acc.cgi?acc=GSE154863]), and converted into Seurat object.

Human research participants

Policy information about studies involving human research participants and Sex and Gender in Research.

Reporting on sex and gender	Serum samples were obtained from 22 females (4 benign tumors and 18 CRCs) and 34 males (15 benign tumors and 19 CRCs). TMAs of 331 surgically resected human CRC samples were obtained from males (n = 169) and females (n = 162) (age: 21-96 (median = 68)) at Osaka Metropolitan University Hospital.
Population characteristics	Serum samples were obtained from 37 patients (sex, male: n = 19, female: n = 18; age: 36-91 (median = 72); stage, stage 0: n = 5, stage 1: n = 12, stage 2: n = 8, stage 3: n = 12) with CRCs and 19 patients with benign tumors (sex, male: n = 15, female: n = 4; age: 57-89 (median = 72); histology, adenoma: n = 15, sessile serrated lesion: n = 4). All patients underwent surgical or endoscopic resection. TMAs of surgically resected human CRC samples were obtained from 331 patients (sex, male: n = 169, female: n = 162; age: 21-96 (median = 68); stage, stage 2: n = 180, stage 3: n = 151) at Osaka Metropolitan University Hospital.
Recruitment	For collecting serum samples, patients with CRCs or benign tumors who underwent surgical or endoscopic resection in the period between July 2021 and June 2022 were recruited. Patients with stage 4 CRCs were excluded because most of them took chemotherapy and did not undergo surgical resection. For collecting TMA samples, patients with CRCs who underwent surgical resection in the period between January 2007 and December 2012 at Osaka Metropolitan University Hospital were recrutted.
Ethics oversight	Written informed consent was obtained from all patients who provided serum samples with the protocol approved by the Ethics Committee of Kyoto University Graduate School and Faculty of Medicine. The Ethics Committee of Kyoto University and Osaka Metropolitan University approved the use of patient TMA samples for this experiment without requiring written informed consent. Informed consent was obtained in the form of opt-out on the web-site.

Note that full information on the approval of the study protocol must also be provided in the manuscript.

Field-specific reporting

Please select the one below that is the best fit for your research. If you are not sure, read the appropriate sections before making your selection.

[x] Life sciences [ ] Behavioural & social sciences [ ] Ecological, evolutionary & environmental sciences

For a reference copy of the document with all sections, see nature.com/documents/nr-reporting-summary-flat.pdf

Life sciences study design

All studies must disclose on these points even when the disclosure is negative.

Sample size	The sample size chosen for our animal experiments in this study was determined by G*Power Data analysis of two-tailed t-test between 2 groups (α=0.05, power (1-β)=0.8). Based on prior experience of our laboratory performing similar sets of experiments (Nakanishi et al. Cell Reports, 16 3297-3310 (2016)), effect size "d" was calculated at more than "2", and thus the required sample size was "12" in total (n = 6 for each group). For the other experiments, previously published results, complexity, and past experience were used to determine the sample size. Reproducibility between biological replicates and independent experiments as well as the magnitude and consistency of measurable differences between groups was also considered.
Data exclusions	We have not excluded any data.

Replication	Animal experiments were independently performed for at least two times with more than two replicated. In vitro data have been performed independently for at least three times with three replicated. All attempts at replication were successful.
Randomization	Sex- (male) and age- (8-10 weeks old) matched animals were randomly allocated from each genotype into experimental groups. In vitro studies, cells or conditions were assigned randomly to each experimental group.
Blinding	An identification code was assigned to each animal and the investigators were not blinded to group allocation at the time of data collection and analysis.

Reporting for specific materials, systems and methods

We require information from authors about some types of materials, experimental systems and methods used in many studies. Here, indicate whether each material, system or method listed is relevant to your study. If you are not sure if a list item applies to your research, read the appropriate section before selecting a response.

Materials & experimental systems

n/a	Involved in the study	
☐	☒	Antibodies
☐	☒	Eukaryotic cell lines
☒	☐	Palaeontology and archaeology
☐	☒	Animals and other organisms
☒	☐	Clinical data
☒	☐	Dual use research of concern

Methods

n/a	Involved in the study	
☒	☐	ChIP-seq
☐	☒	Flow cytometry
☒	☐	MRI-based neuroimaging

Antibodies

Antibodies used	For immunohistochemistry or immunofluorescence, antibodies used; THBS1 (MA5-13398; Invitrogen), CD14 (75181; Cell Signaling), CD8 (85336; Cell Signaling), EPCAM (ab71916; Abcam), NLRP3 (NBP2-12446; Novus Bio), MLH1 (47954; Cell Signaling), PMS2 (556415; BD Biosciences), MSH2 (NA27; Calbiochem), MSH6 (610919; BD Biosciences), CD31 (7769; Cell Signaling), CD8 (ab209775; Abcam), C-Cas3 (9664; Cell Signaling), CK19 (ab52625; Abcam), CD11b (ab133357; Abcam), GFP (ab6673; Abcam), αSMA (ab5694; Abcam), pSMAD3 (ab52903; Abcam), CD68 (76437; Cell Signaling), CD4 (ab183685; Abcam), CD11c (97585; Cell Signaling), Ly6C (128002; Biolegend), Ly6G (87048; Cell Signaling), F4/80 (70076; Cell Signaling), FOXP3 (12653; Cell Signaling), TCF7 (MA514965; Thermo Fisher), CD36 (ab202909; Abcam), CD47 (SC-53050; Santa Cruz), CXCR4 (ab181020; Abcam), CTLA4 (ab237712; Abcam), donkey anti-rabbit IgG secondary antibody (A21206, A21207; Thermo fisher), donkey anti-mouse IgG secondary antibody (A21202, A21203; Thermo fisher), anti-rabbit IgG (BA-1000; Vector Laboratories), anti-goat IgG (BA-5000; Vector Laboratories), anti-rat IgG (BA-4000; Vector Laboratories), anti-mouse IgG (BA-2000; Vector Laboratories), ImmPRESS Reagent (MP-7401, MP-7404; Vector Laboratories), EnVision+ System-HRP Labelled Polymer (K4001; Dako). For flow cytometry, antibodies used; CD45 (552848, 557659; BD Biosciences, 103106; Biolegend), CD11b (553310, BD Biosciences), CXCR4 (146511, BioLegend), EpCAM (48-5791-82, Thermo Fisher), CD3 (555275; BD Biosciences, 100236; Biolegend), CD4 (100406; Biolegend), CD8 (553035; BD Biosciences, 100706; Biolegend), CD69 (104512; Biolegend), CD11c (117318; Biolegend), F4/80 (565410; BD Biosciences), Ly6C (560595; BD Biosciences), Ly6G (562737; BD Biosciences), PDCD1 (135224; Biolegend), CTLA4 (106314; Biolegend), CCR2 (150610; Biolegend), FOXP3 (562996; BD Biosciences), IFNg (563376; BD Biosciences), TCF7 (566692; BD Biosciences) . For immunoblotting, antibodies used; Rabbit anti-mouse phospho-Smad3, clone C25A9 (9520, Cell Signaling Technology), Rabbit anti-mouse polyclonal Smad3 (9513, Cell Signaling Technology), Rabbit anti-mouse βactin, clone AC-15 (A1978, Sigma-Aldrich). For in vivo treatment, antibodies used; InVivoMAb rat anti-mouse CD8α, clone 2.43 (BE0061, Bio X cell), InVivoMAb rat anti-mouse PD-1 (CD279), clone RMP1-14 (BE0146, Bio X cell), InVivoMAb rat anti-mouse VEGFR-2, clone DC101 (BE0060, Bio X cell), InVivoMAb rat IgG2b isotype control, clone LTF-2 (BE0090, Bio X cell), InVivoMAb rat IgG2a isotype control, clone 2A3 (BE0089, Bio X cell).
Validation	All antibodies were commercially available and validated by manufacturer. Manufacturer's validation statements are described on the following websites; THBS1 (MA5-13398; Invitrogen) [https://www.thermofisher.com/antibody/product/Thrombospondin-1-Antibody-clone-A6-1-Monoclonal/MA5-13398], CD14 (75181; Cell Signaling) [https://www.cellsignal.jp/products/primary-antibodies/cd14-d7a2t-rabbit-mab-ihc-formulated/75181], CD8 (85336; Cell Signaling) [https://www.cellsignal.jp/products/primary-antibodies/cd8a-d8a8y-rabbit-mab/85336], EPCAM (ab71916; Abcam) [https://www.abcam.co.jp/products/primary-antibodies/epcam-antibody-ab71916.html], NLRP3 (NBP2-12446; Novus Bio) [https://www.novusbio.com/products/nlrp3-nalp3-antibody_nbp2-12446], MLH1 (47954; Cell Signaling) [https://www.cellsignal.jp/products/primary-antibodies/mlh1-es05-mouse-mab/47954], PMS2 (556415; BD Biosciences) [https://www.bdbiosciences.com/ja-jp/products/reagents/microscopy-imaging-reagents/immunofluorescence-reagents/purified-mouse-anti-pms2.556415], MSH2 (NA27; Calbiochem) [https://www.merckmillipore.com/JP/ja/product/Anti-MSH2-Ab-2-Mouse-mAb-FE11,EMD_BIO-NA27], MSH6 (610919; BD Biosciences) [https://www.bdbiosciences.com/ja-jp/products/reagents/microscopy-imaging-reagents/immunofluorescence-reagents/purified-mouse-anti-msh6.610919], CD31 (7769; Cell Signaling) [https://www.cellsignal.jp/products/primary-antibodies/cd31-pecam-1-d8v9e-xp-rabbit-mab/77699], CD8 (ab209775; Abcam) [https://www.abcam.co.jp/products/primary-antibodies/cd8-alpha-antibody-epr20305-ab209775.html], C-Cas3 (9664; Cell Signaling) [https://www.cellsignal.jp/products/primary-antibodies/cleaved-caspase-3-asp175-5a1e-rabbit-mab/9664], CK19 (ab52625; Abcam) [https://www.abcam.co.jp/products/primary-antibodies/cytokeratin-19-antibody-ep1580y-cytoskeleton-marker-ab52625.html], CD11b (ab133357; Abcam) [https://www.abcam.co.jp/products/primary-antibodies/cd11b-antibody-epr1344-ab133357.html], GFP (ab6673; Abcam) [https://www.abcam.co.jp/products/primary-antibodies/gfp-antibody-ab6673.html], αSMA (ab5694; Abcam) [https://www.abcam.co.jp/products/primary-antibodies/alpha-smooth-muscle-actin-antibody-ab5694.html], pSMAD3 (ab52903; Abcam) [https://www.abcam.co.jp/products/primary-antibodies/smad3-phospho-s423--s425-antibody-ep823y-ab52903.html], CD68 (76437; Cell Signaling) [https://www.cellsignal.jp/products/primary-antibodies/cd68-d4b9c-xp-rabbit-

mab/76437], CD4 (ab183685; Abcam) [https://www.abcam.co.jp/products/primary-antibodies/cd4-antibody-epr19514-ab183685.html], CD11c (97585; Cell Signaling) [https://www.cellsignal.jp/products/primary-antibodies/cd11c-d1v9y-rabbit-mab/97585], Ly6C (128002; Biolegend) [https://www.biolegend.com/ja-jp/explore-new-products/purified-anti-mouse-ly-6c-antibody-4894], Ly6G (87048; Cell Signaling) [https://www.cellsignal.jp/products/primary-antibodies/ly-6g-e6z1t-rabbit-mab/87048], F4/80 (70076; Cell Signaling) [https://www.cellsignal.jp/products/primary-antibodies/f4-80-d2s9r-xp-rabbit-mab/70076], FOXP3 (12653; Cell Signaling) [https://www.cellsignal.jp/products/primary-antibodies/foxp3-d6o8r-rabbit-mab/12653], TCF7 (MA514965; Thermo Fisher) [https://www.thermofisher.com/antibody/product/TCF7-Antibody-clone-C-725-7-Monoclonal/MA5-14965], CD36 (ab202909; Abcam) [https://www.abcam.com/products/primary-antibodies/cd69-antibody-ab202909.html], CD47 (SC-53050; Santa Cruz) [https://www.scbt.com/ja/p/cd47-antibody-ox101], CXCR4 (ab181020; Abcam) [https://www.abcam.co.jp/products/primary-antibodies/cxcr4-antibody-epumbr3-ab181020.html], CTLA4 (ab237712; Abcam) [https://www.abcam.co.jp/products/primary-antibodies/ctla4-antibody-cal49-ab237712.html], donkey anti-rabbit IgG secondary antibody (A21206; Thermo fisher) [https://www.thermofisher.com/antibody/product/Donkey-anti-Rabbit-IgG-H-L-Highly-Cross-Adsorbed-Secondary-Antibody-Polyclonal/A-21206], donkey anti-rabbit IgG secondary antibody (A21207; Thermo fisher) [https://www.thermofisher.com/antibody/product/Donkey-anti-Rabbit-IgG-H-L-Highly-Cross-Adsorbed-Secondary-Antibody-Polyclonal/A-21207], donkey anti-mouse IgG secondary antibody (A21202; Thermo fisher) [https://www.thermofisher.com/antibody/product/Donkey-anti-Mouse-IgG-H-L-Highly-Cross-Adsorbed-Secondary-Antibody-Polyclonal/A-21202], donkey anti-mouse IgG secondary antibody (A21203; Thermo fisher) [https://www.thermofisher.com/antibody/product/Donkey-anti-Mouse-IgG-H-L-Highly-Cross-Adsorbed-Secondary-Antibody-Polyclonal/A-21203], anti-rabbit IgG (BA-1000; Vector Laboratories) [https://vectorlabs.com/products/antibodies/biotinylated-goat-anti-rabbit-igg], anti-goat IgG (BA-5000; Vector Laboratories) [https://vectorlabs.com/products/antibodies/biotinylated-rabbit-anti-goat-igg], anti-rat IgG (BA-4000; Vector Laboratories) [https://vectorlabs.com/products/antibodies/biotinylated-rabbit-anti-rat-igg], anti-mouse IgG (BA-2000; Vector Laboratories) [https://vectorlabs.com/products/antibodies/biotinylated-horse-anti-mouse-igg], ImmPRESS Reagent (MP-7401; Vector Laboratories) [https://vectorlabs.com/products/enzyme-polymer/immpress-hrp-horse-anti-rabbit-igg], ImmPRESS Reagent (MP-7404; Vector Laboratories) [https://vectorlabs.com/products/enzyme-polymer/immpress-hrp-goat-anti-rat-igg-kit], EnVision+ System-HRP Labelled Polymer (K4001; Dako) [https://www.agilent.com/cs/library/packageinsert/public/105435005.PDF], CD45 (552848; BD Biosciences) [https://www.bdbiosciences.com/ja-jp/products/reagents/flow-cytometry-reagents/research-reagents/single-color-antibodies-ruo/pe-cy-7-rat-anti-mouse-cd45.552848], CD45 (557659; BD Biosciences) [https://www.bdbiosciences.com/ja-jp/products/reagents/flow-cytometry-reagents/research-reagents/single-color-antibodies-ruo/apc-cy-7-rat-anti-mouse-cd45.561037], CD45 (103106; Biolegend) [https://www.biolegend.com/ja-jp/productstab/pe-anti-mouse-cd45-antibody-100], CD11b (553310, BD Biosciences) [https://www.bdbiosciences.com/ja-jp/products/reagents/flow-cytometry-reagents/research-reagents/single-color-antibodies-ruo/fitc-rat-anti-cd11b.553310], CXCR4 (146511, BioLegend) [https://www.biolegend.com/ja-jp/search-results/brilliant-violet-421-anti-mouse-cd184-cxcr4-antibody-9060], EpCAM (48-5791-82, Thermo Fisher) [https://www.thermofisher.com/antibody/product/CD326-EpCAM-Antibody-clone-G8-8-Monoclonal/48-5791-82], CD3 (555275; BD Biosciences) [https://www.bdbiosciences.com/ja-jp/products/reagents/flow-cytometry-reagents/research-reagents/single-color-antibodies-ruo/pe-rat-anti-mouse-cd3-molecular-complex.555275], CD3 (100236; Biolegend) [https://www.biolegend.com/ja-jp/products/apc-anti-mouse-cd3-antibody-8055?GroupID=BLG242], CD4 (100406; Biolegend) [https://www.biolegend.com/ja-jp/clone-search/fitc-anti-mouse-cd4-antibody-248?GroupID=BLG4211], CD8 (553035; BD Biosciences) [https://www.bdbiosciences.com/ja-jp/products/reagents/flow-cytometry-reagents/research-reagents/single-color-antibodies-ruo/apc-rat-anti-mouse-cd8a.553035], CD8 (100706; Biolegend) [https://www.biolegend.com/ja-jp/neuroscience-1/fitc-anti-mouse-cd8a-antibody-153], CD69 (104512; Biolegend) [https://www.biolegend.com/ja-jp/productstab/pe-cyanine7-anti-mouse-cd69-antibody-3168], CD11c (117318; Biolegend) [https://www.biolegend.com/ja-jp/clone-search/pe-cyanine7-anti-mouse-cd11c-antibody-3086?GroupID=BLG11937], F4/80 (565410; BD Biosciences) [https://www.bdbiosciences.com/ja-jp/products/reagents/flow-cytometry-reagents/research-reagents/single-color-antibodies-ruo/pe-rat-anti-mouse-f4-80.565410], Ly6C (560595; BD Biosciences) [https://www.bdbiosciences.com/ja-jp/products/reagents/flow-cytometry-reagents/research-reagents/single-color-antibodies-ruo/apc-rat-anti-mouse-ly-6c.560595], Ly6G (562737; BD Biosciences) [https://www.bdbiosciences.com/ja-jp/products/reagents/flow-cytometry-reagents/research-reagents/single-color-antibodies-ruo/bv421-rat-anti-mouse-ly-6g.562737], PDCD1 (135224; Biolegend) [https://www.biolegend.com/ja-jp/products/apc-cyanine7-anti-mouse-cd279-pd-1-antibody-9742], CTLA4 (106314; Biolegend) [https://www.biolegend.com/ja-jp/search-results/pe-cyanine7-anti-mouse-cd152-antibody-10318?Clone=UC10-4B9], CCR2 (150610; Biolegend) [https://www.biolegend.com/ja-jp/global-elements/pdf-popup/pe-anti-mouse-cd192-ccr2-antibody-13336?GroupID=BLG14694], FOXP3 (562996; BD Biosciences) [https://www.bdbiosciences.com/ja-jp/products/reagents/flow-cytometry-reagents/research-reagents/single-color-antibodies-ruo/bv421-rat-anti-mouse-foxp3.562996], IFNg (563376; BD Biosciences) [https://www.bdbiosciences.com/ja-jp/products/reagents/flow-cytometry-reagents/research-reagents/single-color-antibodies-ruo/bv421-rat-anti-mouse-ifn.563376], TCF7 (566692; BD Biosciences) [https://www.bdbiosciences.com/ja-jp/products/reagents/flow-cytometry-reagents/research-reagents/single-color-antibodies-ruo/bv421-mouse-anti-tcf-7-tcf-1.566692], Rabbit anti-mouse phospho-Smad3, clone C25A9 (9520, Cell Signaling Technology) [https://www.cellsignal.jp/products/primary-antibodies/phospho-smad3-ser423-425-c25a9-rabbit-mab/9520], Rabbit anti-mouse polyclonal Smad3 (9513, Cell Signaling Technology) [https://www.cellsignal.jp/products/primary-antibodies/smad3-antibody/9513], Rabbit anti-mouse βactin, clone AC-15 (A1978, Sigma-Aldrich) [https://www.sigmaaldrich.com/JP/ja/product/sigma/a1978], InVivoMAb rat anti-mouse CD8α, clone 2.43 (BE0061, Bio X cell) [https://bioxcell.com/invivomab-anti-mouse-cd8a-be0061], InVivoMAb rat anti-mouse PD-1 (CD279), clone RMP1-14 (BE0146, Bio X cell) [https://bioxcell.com/invivomab-anti-mouse-pd-1-cd279-be0146], InVivoMAb rat anti-mouse VEGFR-2, clone DC101 (BE0060, Bio X cell) [https://bioxcell.com/invivomab-anti-mouse-vegfr-2-be0060], InVivoMAb rat IgG2b isotype control, clone LTF-2 (BE0090, Bio X cell) [https://bioxcell.com/invivomab-rat-igg2b-isotype-control-anti-keyhole-limpet-hemocyanin-be0090], InVivoMAb rat IgG2a isotype control, clone 2A3 (BE0089, Bio X cell) [https://bioxcell.com/invivomab-rat-igg2a-isotype-control-anti-trinitrophenol-be0089].

Eukaryotic cell lines

Policy information about cell lines and Sex and Gender in Research

Cell line source(s)	Mouse tumor organoids (MTO); kindly provided by Dr. Battle's laboratory, MC38; kindly provided by Dr. Honjo's laboratory, C166; purchased from ATCC (# CRL-2581), OVA53; kindly provided by Dr. Takehito Sato.
Authentication	None of the cell lines was authenticated.
Mycoplasma contamination	The cell lines were not tested for mycoplasma contamination.

Commonly misidentified lines (See ICLAC register)	No commonly misidentified cell lines were used.

Animals and other research organisms

Policy information about studies involving animals; ARRIVE guidelines recommended for reporting animal research, and Sex and Gender in Research

Laboratory animals	Thbs1-deficient (Thbs1-/- (#006141)), LysM-Cre (#004781) and Rosa26-EYFP (#006148) mice were purchased from Jackson Laboratories. C57BL/6 mice, GFP transgenic mice (C57BL/6-Tg (CAG-EGFP)) mice were purchased from Japan SLC,Inc. Systemic CD47-deficient (CD47-/-) (CD47 f/f;E2A-Cre) mice were generated by crossing CD47f/f mice with E2A-Cre mice (Saito, Y. et al. SIRPα. PNAS 114, E10151-E10160 (2017)). Thbs1f/f mice were generated as previously described (Yang, H et al. Arterioscler Thromb Vasc Biol 40, e350-e366 (2020)). CD36-deficient (CD36-/-) mice were kindly provided by Dr. Freeman (Harvard Medical School). All mouse strains were generated in a C57BL/6 background and were born and maintained under pathogen-free conditions. These mice were maintained in 14hr light / 10hr dark cycle, and the housing temperature and humidity were maintained were 24°C and 50%, respectively. 8-10 weeks old male mice were used in all experiments.
Wild animals	This study did not involve wild animals.
Reporting on sex	Only male mice were used in this study.
Field-collected samples	This study did not involve samples collected from field.
Ethics oversight	All mouse strains were generated in a C57BL/6 background and were born and maintained under pathogen-free conditions. Animal handling and experimental procedures conformed to institutional guidelines and were approved by the animal research committee of Kyoto University (Kyoto, Japan) and performed in accordance with Japanese government regulations. Male mice were used in all experiments.

Note that full information on the approval of the study protocol must also be provided in the manuscript.

Flow Cytometry

Plots

Confirm that:

- [x] The axis labels state the marker and fluorochrome used (e.g. CD4-FITC).
- [x] The axis scales are clearly visible. Include numbers along axes only for bottom left plot of group (a 'group' is an analysis of identical markers).
- [x] All plots are contour plots with outliers or pseudocolor plots.
- [x] A numerical value for number of cells or percentage (with statistics) is provided.

Methodology

Sample preparation	Flow cytometry experiments were performed on tumor cells isolated from orthotopic tumors, bone marrow cells and blood cells. Briefly, fresh tumors were harvested, rinsed in cold PBS, minced into small pieces, and dissociated with Tumor Dissociation Kit (Miltenyi Biotec). Red blood cells were removed with using Red Blood Cell Lysis Solution (Miltenyi Biotec) and samples were resuspended in PBS supplemented with 1% FBS.
Instrument	Flow cytometry was performed on a FACSAria Ⅱ (BD Biosciences).
Software	Data were collected using BD FACSDiva software and analyzed using FlowJo v10.
Cell population abundance	Cell population data were collected on a debris exclusion gate at the time of acquisition of BD FACSDiva software.
Gating strategy	For all experiments, all cells were gated by FSC area vs. SSC area, and singlets were gated by FSC height vs FSC width and SSC height vs SSC width. For analysis of stimulation of T cells, gated cells were further gated to CD3+CD8+CD69+, CD3+CD8+IFNg+, CD3+CD8+TCF7+ for activated CD8+ T cells, and CD3+CD8+PDCD1+, CD3+CD8+CTLA4+ for exhausted CD8+ T cells. For analysis of immune cells in orthotopic tumors, gated cells were further gated to CD45+CD3+CD4+ for CD4+ T cells, CD45+CD3+CD8+ for CD8+ T cells, CD45+CD3+FOXP3+ for Treg, CD45+CD11c+ for dendritic cells, CD11b+F4/80+ for TAM, CD45+CD11c-CD11b+Ly6C+Ly6G+F4/80- for neutrophil, CD45+CD11c-CD11b+Ly6C+Ly6G-F4/80- for monocyte, CD45+CD11c-CD11b+Ly6C+Ly6G+ for PMN-MDSC, CD45+Cd11c-CD11b+Ly6C+Ly6G- for MO-MDSC, CD45+CD3+CD8+PDCD1+ or CD45+CD3+CD8+CTLA4+ for exhausted CD8+ T cell. For analysis of THBS1-expressing cells, gated cells were further gated on THBS1, CD45, CD11b, CXCR4, Ly6C and CCR2. For analysis of GFP-BM chimeric mice tumors, gated cells were further gated on EPCAM, GFP. For analysis of bone marrow cells, gated cells were further gated on CD45,CD11b.. For analysis of peripheral blood, gated cells were further gated on CD45, CD11b. Fluorophores were chosen to minimize spectral overlap.

- [x] Tick this box to confirm that a figure exemplifying the gating strategy is provided in the Supplementary Information.
